# Virtualized clinical studies to assess the natural history and impact of gut microbiome modulation in non-hospitalized patients with mild to moderate COVID-19 a randomized, open-label, prospective study with a parallel group study evaluating the physiologic effects of KB109 on gut microbiota structure and function: a structured summary of a study protocol for a randomized controlled study

**DOI:** 10.1186/s13063-021-05157-0

**Published:** 2021-04-02

**Authors:** John P. Haran, Jose C. Pinero, Yan Zheng, Norma Alonzo Palma, Mark Wingertzahn

**Affiliations:** 1 Department of Emergency Medicine, Department of Microbiology and Physiological Systems, Clinical Director of the Center for Microbiome Research, University of Massachusetts Medical School, UMass Memorial Medical Group, Worcester, MA USA; 2 Hope Clinical Trials, Miami, FL USA; 3 Kaleido Biosciences, Inc, Lexington, MA USA

**Keywords:** COVID-19, randomised controlled study, protocol, KB109, microbiome, short-chain fatty acids

## Abstract

**Objectives:**

These 2 parallel studies (K031 and K032) aim to evaluate the safety of KB109 in addition to supportive self-care (SSC) compared with SSC alone in outpatients with mild to moderate coronavirus disease 2019 (COVID-19). KB109 is a novel synthetic glycan that was formulated to modulate the gut microbiome composition and metabolic output in order to increase beneficial short-chain fatty acid (SCFA) production in the gut. The K031 study is designed to evaluate the safety of KB109 and characterize its impact on the natural progression of COVID-19 in patients with mild to moderate disease. The K032 study is evaluating the effect of KB109 on the gut microbiota structure and function in this same patient population. Additionally, both studies are evaluating measures of health care utilization, quality of life (QOL), laboratory indices, biomarkers of inflammation, and serological measures of immunity in patients who received SSC alone or with KB109.

Noteworthy aspects of these outpatient studies include study design measures aimed at limiting in-person interactions to minimize the risk of infection spread, such as use of online diaries, telemedicine, and at-home sample collection.

**Study design:**

K031 and K032 are randomized, controlled, open-label, clinical food studies.

**Participants:**

Inclusion Criteria:

• Adults ≥18 years of age

• Patients willing and able to give informed consent

• Screening/randomization telemedicine visit within 2 days of testing positive test for COVID-19

○ In K031 study, symptomatic patients at COVID-19 testing must report new or worsening symptoms at baseline that have not been present for more than 5 days

▪ Cardinal COVID-19 symptoms include fever, chills/repeated shaking with chills, cough, shortness of breath, headache, muscle pain, anosmia/ageusia, and sore throat. The 5 additional symptoms include gastrointestinal (GI) disturbance/symptoms (other than diarrhea), diarrhea, fatigue, nasal congestion, and chest tightness

○ In K031, at COVID-19 testing, pre-symptomatic patients must report new cardinal COVID-19 symptoms within 7 days of a positive test and they must be screened and randomized within 5 days of developing symptoms

• Mild to moderate COVID-19 and self-reported outpatient management

○ In K032, mild to moderate COVID-19 was defined as having the following symptoms for no more than 72 hours before COVID-19 testing: a self- reported fever or cough (new or exacerbated) or presence of at least 2 of the following: anosmia, sore throat, or nasal congestion

• Ability to adhere to the study visit schedule and other protocol requirements

• Consistent internet or cell phone access with a data plan and access to a smartphone, tablet, or computer

• The K031 and K032 studies are currently being conducted at 17 clinical institutions throughout the United States.

Exclusion Criteria:

• In the primary investigator’s (PI) judgement, patients likely to require hospitalization for COVID-19

• Patients who are hospitalized for in-patient treatment or currently being evaluated for potential hospitalization at the time of informed consent for conditions other than COVID-19

• History of chronic lung disease with chronic hypoxia

• History of documented cirrhosis or end-stage liver disease

• Ongoing requirement for oxygen therapy

• Shortness of breath in resting position

• Diagnosis of sleep apnea requiring bilevel positive airway pressure (BIPAP)/continuous positive airway pressure (CPAP)

• Female patients who are pregnant, trying to become pregnant, or lactating

• Concurrent use of immunomodulatory agent within 12 months; systemic antibiotics, antifungals, or antivirals for treatment of active infection within 28 days; systemic immunosuppressive therapy within 3 months; or drugs or other compounds that modulate GI motility (eg, stool softeners, laxatives, or fiber supplements) taken currently, or within 7 days. Antacid (histamine 2 blockers and proton pump inhibitors) and antidiarrheal agents are not prohibited

• History of GI surgery (6 months prior to randomization), including but not limited to bariatric surgery and bowel resection, or history of, or active GI disease(s) that may affect assessment of tolerability, including but not limited to inflammatory bowel disease, irritable bowel syndrome, autoimmune disease, or GI malignancy

• Participation in an interventional clinical trial or use of any investigational agent within 30 days before randomization

• Clinically significant or uncontrolled concomitant medical condition that would put the patient at risk or jeopardize the objectives of the study in the opinion of the PI

• In the opinion of the PI, patient unlikely for any reason to be able to comply with study procedures

• Contraindications, sensitivities, or known allergy to the use of the study product or its components

**Intervention and comparator:**

Patients will be randomized (1,1) to receive either SSC and KB109 or SSC alone. During SSC, patients should follow the steps as instructed by their healthcare provider to care for themselves and protect other people in the home and community from potentially contracting COVID-19. Management of COVID-19-related symptoms with over-the-counter cough, cold, and anti-pyretic medications by patients is permitted in accordance with the medications’ respective drug facts label or as instructed by the patient’s healthcare provider.

Following randomization, patients assigned to receive KB109 and SSC will receive a Kaleido Biosciences, Inc at-home study kit including a thermometer, pulse oximeter, and KB109.

During the Intake Period (days 1–14), KB109 will be reconstituted in water by the patient and consumed by the patient twice daily (at least 8 hours apart), following an up-titration dosing schedule:

Days 1 to 2: 9 g twice daily for a total daily dose of 18 g Days 3 to 4: 18 g twice daily for a total daily dose of 36 g Days 5 to 14: 36 g twice daily for a total daily dose of 72 g

During the intake period, patients will record their daily COVID-19–related symptoms, selected COVID-19 signs (as self-measured using the provided thermometer and pulse oximeter), responses to questions related to QOL measures, health care use measures, and concomitant medications taken in the previous 24 hours. Wellness visits by telephone will be conducted between days 1 and 14 to follow up on patient’s health status and to ascertain compliance with KB109 and completion of questions. On day 14, all patients will undergo a telemedicine visit where the following will be conducted: abbreviated physical examination, assessment of safety and other protocol-specified measures of health, and an evaluation of whether follow-up treatment is recommended owing to a progression of COVID-19 symptoms. If feasible, blood samples for clinical chemistries, biomarkers and serological measure of immunity, and nasal/oropharyngeal swabs for quantitative viral load assessments will be collected.

Beginning on day 15, patients in both groups will enter the follow-up period (days 15–35) where COVID-19 signs, symptoms, and health care use indices will be collected. Wellness visits by telephone will be conducted on days 21, 28, and 35 to follow-up on the patient’s health status. On day 35, all patients will undergo a telemedicine visit where the same information as the day 14 telemedicine visit will be collected, including any blood samples.

**Main outcomes:**

The primary outcome for the K031 and K032 studies is to evaluate the safety of KB109 in addition to SSC compared with SSC alone in outpatients with mild to moderate COVID-19 by assessing the number of patients experiencing KB109-related treatment-emergent adverse events (TEAEs) during the study.

K031 will also evaluate duration of symptoms among outpatients with mild to moderate COVID-19. This will be as an assessment made during the intake and/or follow-up periods of the following:

• Time to resolution of the 13 overall and the 8 cardinal COVID-19–related symptoms from day 1 until the day at which the composite score of the 13 overall and 8 cardinal COVID-19–related symptoms becomes 0 or 1 and remains at 0 or 1 for the rest of the intake period and for the follow-up period

• Proportion of patients with a reduction from baseline in each of the 13 overall COVID-19–related symptoms

• Proportion of patients in whom symptoms (present at baseline) become absent for each of the 13 overall COVID-19–related symptoms

• Change from baseline in the overall composite score of the 13 overall COVID-19–related symptoms and the 8 cardinal COVID-19–related symptoms

• Time to resolution of fever (defined as from day 1 until the day at which a patient’s daily maximum temperature achieves and remains below 100.4°F without antipyretic medication)

• Proportion of patients with oxygen saturation <95% and <98% on days 14 and 35

• Measures collected from the health care provider wellness visits

• Proportion of patients experiencing hospital admissions (all cause and COVID-19–related)

• Health care use

K032 will evaluate the effect of KB109 in addition to SSC compared with SSC alone on the gut microbiota structure and function in outpatients with mild to moderate COVID-19. Before days 1, 14, and 35, microbiota structure (eg, magnitude of change in gut microbiome structure, composition of gut microbiome) will be analysed by methods such as nucleic acid sequencing and gut microbiome function will be analysed via levels of stool inflammatory biomarkers (eg, lipocalin) and gut microbiome metabolites (eg, SCFA). The health of outpatients with mild to moderate COVID-19 will be evaluated during the intake and follow- up periods by: measures of QOL; measures collected from the healthcare provider wellness visits; the proportion of patients experiencing hospital admissions; health care use, the proportions of patients with oxygen saturation <95% and <98%, and the proportion of patients with temperature below 100.4 °F without an anti-pyretic medication. Potential exploratory outcome measures may include: changes from baseline (day 1) in laboratory measures, specific biomarkers of infection, serology, inflammation (eg, D-dimer, lipocalin, cytokines, IgM/IgG sero-conversion, and neutralization assays), and viral load in outpatients with mild to moderate COVID-19 in the presence and absence of KB109.

**Randomisation:**

All patients deemed eligible for the studies will be randomized in a 1:1 ratio to KB109 in addition to SSC or SSC alone group using an interactive response technology system. Randomization will be stratified by study site/center, age groups (≥18–<45 years, ≥45–<65 years, ≥65 years), and comorbidity status (yes, no).

**Blinding (masking):**

These studies are open-label; therefore, no blinding is necessary.

**Numbers to be randomised (sample size):**

K031 will enroll approximately 350 to 400 (175–200 patients per group) whereas K032 will enroll approximately 50 patients (25 per group).

**Study status:**

K031 protocol version 4, December 9, 2020; recruitment started in August, 2020, and the study is estimated to be completed in March 2021. This study is active and enrollment was completed in January, 2021.

K032 protocol version 2, June 30, 2020; recruitment is estimated to start in July, 2020. This study is recruiting and the study is estimated to be completed in March 2021.

**Study registration:**

K031 is registered with the US National Library of Medicine, Identifier NCT04414124 as of June 4, 2020.

K032 is registered with the US National Library of Medicine, Identifier NCT04486482 as of July 24, 2020.

**Full protocol:**

The full protocols are attached as additional files (Additional files 1 and 2), accessible from the ClinicalTrials.gov website. In the interest in expediting dissemination of this material, the familiar formatting has been eliminated; this letter serves as a summary of the key elements of the full protocols.

The study protocols have been reported in accordance with the Standard Protocol Items: Recommendations for Clinical Interventional Trials (SPIRIT) guidelines (Additional files 3 and 4).

**Supplementary Information:**

The online version contains supplementary material available at 10.1186/s13063-021-05157-0.

## Supplementary Information

**Additional file 1.**

**Additional file 2.**

**Additional file 3.**

**Additional file 4.**

## Data Availability

Qualified scientific and medical researchers may make requests for individual participant data that underlie the results (text, tables, figures, and supplement) reported in this article, after de-identification, at clinicalstudies@kaleido.com. Methodologically sound proposals for such data will be evaluated and approved by Kaleido Biosciences, Inc, in its sole discretion. All approved researchers must sign a data access agreement prior to accessing the data. Data will be available as soon as possible but no later than within 1 year of the acceptance of the article for publication, and for 3 years following article publication. Kaleido Biosciences, Inc, will not share identified participant data or a data dictionary**.**

