# Supplementary material for: Virtualized clinical studies to assess the natural history and impact of gut microbiome modulation in non-hospitalized patients with mild to moderate COVID-19 a randomized, open-label, prospective study with a parallel group study evaluating the physiologic effects of KB109 on gut microbiota structure and function: a structured summary of a study protocol for a randomized controlled study
Source: Trials. 2021 Apr 2;22:245. doi: 10.1186/s13063-021-05157-0 (PMC8017509; doi:10.1186/s13063-021-05157-0)
Supplement: Supplementary file 1 — Additional file 1. [file 13063_2021_5157_MOESM1_ESM.pdf]

An Exploratory, Open Label, Clinical Study to Evaluate the Physiologic Effects of KB109 in Adult Patients with Mild-to-Moderate COVID-19 on Gut Microbiota Structure and Function in the Outpatient Setting

Protocol #: K032-120

Sponsor: Kaleido Biosciences, Inc.
65 Hayden Ave
Lexington, MA 02421, USA

Version #: 2

Version Date: 30 June 2020

This document is a confidential communication of Kaleido Biosciences, Inc. (Kaleido). The recipient agrees that no information contained herein will be published or disclosed without Kaleido's prior written approval, except that this document may be disclosed to appropriate institutional review boards or duly authorized representatives of the appropriate regulatory agencies under the condition they are requested to keep it confidential.

SIGNATURE OF PRINCIPAL INVESTIGATOR

Study Title: An Exploratory, Open Label, Clinical Study to Evaluate the Physiologic Effects of KB109 in Adult Patients with Mild-to-Moderate COVID-19 on Gut Microbiota Structure and Function in the Outpatient Setting

Protocol Number: K032-120

This protocol is sponsored by Kaleido Biosciences, Inc. (Kaleido). All the data and any other information generated from the execution of this protocol are the sole property of Kaleido and may not be used for any purpose without written permission from Kaleido.

The signature below constitutes the approval of this protocol and provides the necessary assurance that this trial will be conducted according to all stipulations of the protocol, including all statements regarding confidentiality, and according to applicable US Federal regulations, and ICH/GCP guidelines.

Principal Investigator’s Signature: ____________________________________

Principal Investigator’s Name: ____________________________________

Institution Name: ____________________________________

Date: ____________________________________

SPONSOR APPROVAL PAGE

Study Title: An Exploratory, Open Label, Clinical Study to Evaluate the Physiologic Effects of KB109 in Adult Patients with Mild-to-Moderate COVID-19 on Gut Microbiota Structure and Function in the Outpatient Setting

Original Protocol: 30 June 2020

Person authorized to sign the protocol and protocol amendment(s) for the Sponsor, Kaleido:

Sponsor Signature: ______________________

Mark Wingertzahn, PhD
VP Research and Development and Head of Clinical Development
Kaleido Biosciences, Inc.

Date: 1/July/2020

1 PROTOCOL SYNOPSIS

**Name of Sponsor/Company:**<br>Kaleido Biosciences, Inc.
**Name of Finished Product:**<br>Study product (SP) KB109 (spray-dried, synthetic oligosaccharide food composition)
**Name of Substance Investigated:**<br>KB109
**Title of Study:**<br>An Exploratory, Open Label, Clinical Study to Evaluate the Physiologic Effects of KB109 in Adult Patients with Mild-to-Moderate COVID-19 on Gut Microbiota Structure and Function in the Outpatient Setting
**Study Rationale:**<br><br>The COVID-19 pandemic threatens individuals, societies and healthcare systems around the world. Host immunity contributes to the susceptibility as well as the progression of infectious disease and patient outcomes. Clinical studies and *ex vivo* experiments using human fecal samples have demonstrated that commensal bacteria selectively ferment proprietary oligosaccharide mixtures to promote their growth and produce metabolites such as the short-chain fatty acids (SCFAs) including acetic, propionic, and butyric acid. SCFAs modulate host inflammation, control adaptive immunity, and promote immune tolerance in the gut as well as systemically (Thaiss 2016; Schirmer 2016). In particular SCFAs and SCFA-producing taxa have been linked to reduced risk of acquiring viral infections (including corona-viral infections) in at-risk populations (e.g., Hematopoietic stem cell transplantation [HSCT] patients, Haak 2018). Studies in mice have demonstrated that SCFAs and SCFA-producing microbial taxa induce virus-specific CD4+ and CD8+ T cells, interferon type 1, and antibody responses involved in the reduction of viral infection severity (Ichinohe 2011; Trompette 2018). Data has also shown that protection against Respiratory Syncytial Virus (RSV) infection can be conferred by acetate, a metabolite produced by the intestinal microbiota, through induction of interferon (IFN)-β in the lung through GPR43 and IFNAR-mediated pathways (Antunes 2019). In addition, SCFAs have also been reported to influence macrophage functionality to mitigate neutrophil-mediated tissue damage (Trompette 2018). This is of particular importance as viral infections may be accompanied by an aggressive pro-inflammatory response (Haak 2018) that can elicit a syndrome known as "cytokine storm".<br><br>Kaleido's orally administered proprietary oligosaccharide mixtures are related to substances found in the human diet that modulate the composition and metabolic output of the microbiome and are generally recognized as safe (GRAS). They allow the controlled production of SCFAs with respect to absolute quantities as well as in relative abundance. Capitalizing on this property, Kaleido has selected a proprietary oligosaccharide mixture, termed KB109**,** that holds promise in inducing a beneficial profile of SCFA production in the gut while driving the growth of commensal bacteria. Increased SCFA and the modulation in microbiome taxonomy may lead to a more appropriate immune and inflammatory response and may prevent an over-aggressive response, up to and including "cytokine storm" observed in patients with COVID-19.<br><br>KB109 is a chemically synthesized oligosaccharide mixture resulting from the polymerisation of glucose:galactose:mannose that has been studied in humans and has been determined to be GRAS for its intended use. It is intended for oral administration and is supplied as a powder to be reconstituted in water. In an *ex vivo* model, KB109 increased the amount of SCFAs produced over water control by approximately three-fold across multiple healthy fecal communities. In addition, it is currently being assessed in an ongoing clinical food study in patients colonized with multidrug-resistant (MDR) organisms to reduce the relative abundance of associated taxa including *Enterobacteriaceae*, *Enterococcus*, and *C. difficile* compared to observational controls (NCT 03944369).<br><br>In this study, outpatients with mild-to-moderate COVID-19 infection in the presence and absence of KB109 will be evaluated for safety, physiologic effects, and gut microbiota structure and function. Physiologic effects will be assessed by measuring biomarkers of inflammation and antibodies as well as physiologic responses of health in patients, quality of life (QOL), and hospital utilization. Given the novel outpatient design, in an emerging disease, this study will also enable assessment of various feasibility indices to inform future clinical trials in COVID-19.

Study Period: Up to 37 days (including the Screening period through end of assessments)

**Objectives:**	**Endpoint:**
To evaluate the safety of KB109 in addition to Supportive Self Care (SSC + KB109) in outpatients with mild-to-moderate COVID-19 in the presence and absence of KB109	• Number of patients experiencing SP-related treatment-emergent adverse events (TEAEs)
To evaluate the gut microbiota structure in outpatients with mild-to-moderate COVID-19 in the presence and absence of KB109	• Gut microbiota structure (e.g., magnitude of change in gut microbiome structure, composition of gut microbiome) by methods such as nucleic acid sequencing prior to Day 1, and at Day 14 (End of Intake Period, EOI) and Day 35
To evaluate measures of gut microbiome function in outpatients with mild-to-moderate COVID-19 in the presence and absence of KB109	• Levels of stool inflammatory biomarkers (e.g., lipocalin) and gut microbiome metabolites (e.g., SCFA) prior to Day 1, and on Day 14 (EOI) and Day 35
To evaluate measures of health in outpatients with mild-to-moderate COVID-19	• Measures of Quality of Life (QOL)<br>• Measures collected from the Healthcare Provider Wellness Visits<br>• Proportion of patients experiencing hospital admissions during the Intake Period and Follow-up Period<br>• Healthcare Utilization during the Intake Period and Follow-up Period<br>• Proportion of patients with oxygen saturation <95%<br>• Proportion of patients with oxygen saturation <98%<br>• Proportion of patients with temperature below 100.4 °F without an anti-pyretic medication
To evaluate the changes in laboratory measures, specific biomarkers, serology and viral load in outpatients with mild-to-moderate COVID-19 in the presence and absence of KB109.	• Change from Baseline (Day 1) in:<br>o Laboratory measures<br>o Biomarkers of infection, serology, and inflammation (e.g., D-dimer, lipocalin, cytokines, IgM/IgG sero-conversion, and Neutralization Assays)
To evaluate clinical trial feasibility indices	• Aspects of clinical design and conduct including patient agreement with inclusion and exclusion criteria, acceptance of study-specific procedures, technical aspects of trial conduct and data capture, site resource allocation, recruitment strategies, and subject retention

Methodology:

This is an exploratory, open label clinical food study. It is intended to assess KB109 on safety as well as the structure and function of the gut microbiome, and physiologic effects by measure biomarkers of inflammation and serological measures of immunity, and healthcare utilization (including hospitalizations), laboratory/biochemical indices, patient's wellness, and quality-of-life measures in outpatients who have tested positive with COVID-19, have mild-to-moderate disease, and have been advised to manage their disease at home with SSC under quarantine protocols set forth by the CDC or local ordinances or practices as advised by their healthcare provider.

Patients who attend an outpatient clinic for suspected COVID-19 will be recruited for this study. All patients who enter this study will continue to follow the SSC guidance as instructed by the treating healthcare provider at the outpatient clinic. Note

that during this outpatient study, the interaction between study staff and patients will be minimized to limit risk of infection spread. Patients will be recording responses to study-related questions in a secure website called “TrialPace™ diary” (TrialPace) and may have healthcare professionals visit their home for study procedures and assessments as outlined in the Schedule of Assessments (SOA, **Table 1**).

This study comprises 2 parts.

Part 1: The SARS-CoV-2 test will be ordered by the patient’s treating provider. Potential study subjects may have received a test for COVID-19 at a different clinic than the study site location and may present to the study site for consideration for study enrollment. After informed consent, patients sign into TrialPace using a unique log-in and password; this may occur prior to or after having received the result of the SARS-CoV-2 test that was ordered by their treating healthcare provider. Additionally, blood samples for hematology, chemistry, biomarkers and serological measures of immunity will be drawn, and nasal and oropharyngeal swabs for quantitative vial load assessments may be collected if feasible. Patients who have a positive COVID-19 test result and meet eligibility criteria will enter Part 2 of the study no later than 48 hours after testing positive for COVID-19. Hospital, academic, or industry-based assays will be acceptable for the diagnosis of COVID-19; home-based tests are not accepted for this study.

Part 2: Part 2 is comprised of an Intake Period (Days 1-14) and a Follow-up Period (Days 15-35):

Intake Period (Days 1-14): Eligible patients will be randomized (1:1) to receive either SSC + KB109 or remain on SSC alone. The randomization will be stratified by site/center, age sub-group (≥18 to 45 years, ≥45 to 65 years, ≥65 years) and comorbidity status (Yes/No). Upon confirmation of study eligibility, patients will be provided the SP (KB109, if assigned to that group) and the Kaleido At-home Study Kit (KaSK)that will contain the SP dosing instructions (as applicable), a thermometer, and a pulse oximeter. Upon enrollment, subjects will also receive the telemedicine contact information.

During the Intake Period, patients assigned to SSC+KB109 will self-administer KB109 (twice daily) starting on the morning after receipt of the KaSK according to the schema outlined below in addition to maintaining a stable diet.

Patients will consume KB109 orally after reconstitution in water, twice daily, at least 8 hours apart, according to the following dosing schedule:

**SP Dose Administration Period**	**KB109**
Days 1–2	18 g total daily dose consumed in two divided doses (9 g each dose)
Days 3-4	36 g total daily dose consumed in two divided doses (18 g each dose)
Days 5-14	72 g total daily dose consumed in two divided doses (36 g each dose)

During the Intake Period, patients in both groups (SSC+KB109 or SSC alone) will continue to follow the SSC guidance as instructed by the treating healthcare provider. Patients will also record responses to QOL and healthcare utilization questions using TrialPace for 14 days. For patients in the SSC+KB109 group, Day 1 measures will be recorded prior to the first dose of KB109. For all patients in both groups, temperature and oxygen saturation will be measured and recorded as needed throughout the day prior to taking anti-pyretic medication. Patients will be asked to collect a stool sample prior to the first intake of study product for subjects in the KB109 + SSC group or prior to the morning of Day 1 for subjects in the SSC alone group.

Patients will have wellness visits by telephone on Days 3, 7, 10 and 14 where compliance, safety, and health status will be monitored. If the wellness visit falls on the same day as a telemedicine visit, then the wellness visit assessments will be captured during the telemedicine visit (i.e., there will be no separate wellness visit on Day 14). On the last day of the Intake Period (Day 14), patients will have a follow-up telemedicine visit to assess safety, have blood drawn for clinical chemistries, biomarkers and serological measures of immunity, and may have nasal and oropharyngeal sample collection to measure quantitative viral load. Patients will be asked to provide a stool sample within 2 days of or on Day 14 (i.e., Days 12-14). Procedures are outlined in the SOA (**Table 1**).

Follow-up Period (Days 15-35): During the Follow-up Period, patients will continue to record responses to QOL and healthcare utilization. Patients will have wellness visits on Days 21 and 28 where safety and health status will be monitored. On Day 35, patients will have a follow-up telemedicine visit to assess safety and health status, have blood drawn for clinical chemistries, biomarkers and serological measures of immunity, and may have nasal and oropharyngeal sample collection to measure quantitative viral load. Wellness visit assessments will be captured during the telemedicine visit on Day 35. Patients will be asked to provide a stool sample within 2 days of or on Day 35. Procedures are outlined in the SOA (**Table 1**).

Throughout the study, safety will be monitored by adverse events (AEs). COVID-19 related symptoms of the disease under study will not be classified as treatment-emergent adverse events (TEAE) as long as they are within the normal day-to-day fluctuation or expected progression of the disease, not including hospitalizations, and are part of the clinical data of the disease that is being collected in the study. Any patient experiencing a TEAE, significant worsening of COVID-19 symptoms or intolerable GI symptoms will be evaluated by the principal investigator (PI) or designee via a telemedicine visit and referred as needed for emergent follow-up, or in the case of intolerable GI symptoms for patients in the SSC+KB109 group, for interruption and/or down-titration of SP dose to the previously tolerated level.

Number of patients planned: Approximately 50 (25 patients per group)

Eligibility Criteria:

To be considered for enrollment into this study, each patient must meet **all** of the following Inclusion Criteria:

1. Adults ≥18 years of age
2. Be willing and able to give informed consent, provide medical history, and secondary contact information
3. Screening/Randomization within 2 days of testing positive test for COVID-19
4. Mild-to-moderate COVID-19 as having self-reported fever or cough (new or exacerbated chronic) or presence of at least 2 of the following: anosmia, sore throat, or nasal congestion for not more than 72 hours prior to COVID-19 testing
5. Mild to moderate COVID-19 and self-reported outpatient management indicated by their healthcare provider
6. Able to adhere to the study visit schedule and other protocol requirements
7. Has consistent internet or cellphone access with a data plan and access to a smartphone, tablet or computer

Patients who meet **any** of the following Exclusion Criteria at the Randomization Visit will not be enrolled into the study:

1. In the Primary Investigator's judgement, patients likely to require hospitalization for COVID-19
2. Patients who are hospitalized for in-patient treatment or currently being evaluated for potential hospitalization at the time of informed consent for conditions other than COVID-19
3. History of chronic lung disease with chronic hypoxia
4. History of documented cirrhosis or end-stage liver disease
5. Ongoing requirement for oxygen therapy
6. Shortness of breath in resting position
7. Diagnosis of sleep apnea requiring Bilevel Positive Airway Pressure (BIPAP) / Continuous Positive Airway Pressure (CPAP)
8. Female patients who are pregnant, trying to become pregnant or lactating.
9. Concurrent use of any of the following medications:
    a. Therapy with an immunomodulatory agent within 12 months of study screening
    b. Systemic antibiotics, antifungals, or antivirals for treatment of active infection within 28 days of study screening
    c. Systemic immunosuppressive therapy within 3 months of study screening

d. Drugs or other compounds that modulate GI motility (including stool softeners, laxatives, or fiber supplements) taken currently or within 7 days of Study screening

10. History of GI surgery (6 months prior to Randomization) including but not limited to bariatric surgery and bowel resection, or history of, or active GI disease(s) that may affect assessment of tolerability, including but not limited to the following:
    a. Inflammatory bowel disease
    b. Irritable bowel syndrome
    c. Autoimmune disease
    d. GI malignancy
11. Participation in an interventional clinical trial or use of any investigational agent within 30 days before Randomization
12. Has a clinically significant or uncontrolled concomitant medical condition that would put the patient at risk or jeopardize the objectives of the study in the opinion of the PI
13. Is considered, in the opinion of the PI, to be unlikely for any reason to be able to comply with study procedures
14. Contraindications, sensitivities, or known allergy to the use of the study product or its components

Statistical Methods:

This is an exploratory study; thus, no formal sample size or power calculations are employed. The sample size of 50 randomized patients (25 per group) is chosen for practical reasons.

The analysis of AEs will be carried out on the Safety Analysis Set (Section 8). Summaries will be presented by Medical Dictionary for Regulatory Activities (MedDRA) system organ class and preferred term using frequency counts and percentages by group and overall.

In general, continuous data will be summarized by visit using the following descriptive statistics: sample size, mean, standard deviation, median, minimum value, and maximum value. Categorical and qualitative data will be summarized by visit using frequencies and percentages.

Details of the statistical methods are provided in Section 8. Methods related to exploratory objectives and supportive analyses will be described in a separate document such as Statistical Analysis Plan (SAP) which will be developed and completed prior to database lock.

TABLE OF CONTENTS

1 PROTOCOL SYNOPSIS 4
TABLE OF CONTENTS 9
LIST OF TABLES 13
LIST OF FIGURES 13
LIST OF ABBREVIATIONS 14
2 INTRODUCTION 16
2.1 Background 16
2.1.1 Oligosaccharides and the Gut Microbiome 16
2.1.2 COVID-19 17
2.2 Study Rationale 17
2.3 Benefit/Risk Assessment 22
3 STUDY OBJECTIVES AND ENDPOINTS 22
3.1 Study Objectives 22
3.2 Study Endpoints 23
4 STUDY DESIGN 23
4.1 Overall Design 23
4.2 Study Duration 28
4.3 Study Procedures 28
4.3.1 Patient Demographics and Baseline Characteristics 31
4.3.2 Medical History and Concomitant Medications/Procedures 31
4.3.3 Physical Examination 31
4.3.4 Patient-assessed COVID-19 Signs 32
4.3.5 Quality of Life Indices 32
4.3.6 Healthcare Utilization 33
4.3.7 Laboratory and Inflammatory Biomarkers 34
4.3.7.1 Stool Sample Collection 34
4.3.8 Telemedicine Visit 35
4.3.9 Wellness Visit by Telephone 35
5 STUDY POPULATION 35
5.1 Inclusion Criteria 35

5.2 Exclusion Criteria 36
5.3 Screen Failures 37
5.4 Enrollment 37
5.5 Withdrawal of Patients 37
5.5.1 Handling of Discontinuation of SP and Early Terminations 38
5.5.2 Premature Termination or Suspension of Study 38
5.5.3 Lost to Follow-up 39
6 STUDY PRODUCTS 39
6.1 Description of Study Products 39
6.1.1 KB109 39
6.2 SP Dosing, Preparation and Administration 40
6.3 SP Storage and Accountability 41
6.4 Unblinding 41
6.5 Concomitant Medications/Dietary Supplements 41
6.6 Prohibited Medications/Dietary Supplements 41
6.7 Self-supportive Care 42
6.8 Dose Reductions and Holds 43
6.9 Other Study Restrictions 43
6.10 Randomization, Stratification and Blinding 44
7 ADVERSE EVENT REPORTING 44
7.1 Definitions of AEs, Period of Observation and Recording of AEs 44
7.2 AE Assessments 45
7.2.1 Severity 45
7.3 Causality 45
7.3.1 Outcome Categorization 46
7.3.2 Symptoms of the Disease Under Study 46
7.3.3 Clinical Laboratory and Other Assessments 46
7.3.4 Pregnancy 47
7.4 SAE Procedures 47
7.4.1 SAE definition 47
7.4.2 SAE reporting procedures 48
7.4.3 SAE Collection Timeframe 49
7.4.4 SAE Onset and Resolution Dates 49

7.4.5 Fatal Outcome 49
7.4.6 Reporting to health authorities, independent ethics committee and investigators 49
8 STATISTICAL CONSIDERATIONS 50
8.1 Sample Size 50
8.2 Analysis Endpoints 50
8.3 Analysis Set 50
8.3.1 Safety Analysis Set 50
8.4 Statistical Methods 51
8.4.1 Patient Disposition 51
8.4.2 Demographics and Baseline Characteristics 51
8.4.3 Analyses of Endpoints of Interest 51
8.4.4 Extent of Exposure and Compliance 52
9 SUPPORTING DOCUMENTS AND OPERATIONAL CONSIDERATIONS 52
9.1 Regulatory, Ethical, and Study Oversight Considerations 52
9.1.1 Informed Consent 53
9.1.2 Institutional Review Board/Ethics Committee Review 53
9.1.3 Safety Monitoring Plan 54
9.1.4 Quality Assurance and Quality Control 54
9.1.5 Direct Access to Source Data and Documents 54
9.1.6 Publication 54
9.2 Administrative and Legal Obligations 55
9.2.1 Protocol Amendments 55
9.2.2 Halting of Study 55
9.2.3 Study Termination 55
9.2.4 Study Documentation and Archive 56
9.2.5 Electronic Case Report Forms 56
9.2.6 Record Retention 56
9.2.7 SP Control, Accountability, and Disposition 57
9.2.8 Confidentiality 57
9.2.9 Disclosure of Data 57
9.3 Protocol Deviations 57

9.4 Research Use of Stored Human Samples and Data 58
9.5 Future Use of Stored Samples and Data Derived from Samples................................59
10 REFERENCES 60

LIST OF TABLES

Table 1. **Schedule of Assessments**......29

Table 2. **Clinical Laboratory Tests**......34

Table 3. **Dose Up-titration of the Study Product**......40

LIST OF FIGURES

Figure 1: **Representative KB109 batch SCFA production average across three healthy fecal communities normalized to water control**......19

Figure 2: **Reduction of Enterobacteriaceae in ICU patients' microbiota (spiked with CRE E.coli) incubated with KB109 relative to water control**......21

Figure 3: **CRE and VRE growth on KB109 demonstrated similar growth to water control, suggesting an inability of these pathogens to directly utilize KB109**......21

Figure 4: **Study Design**......28

Figure 5: **KB109 Structure**......40

LIST OF ABBREVIATIONS

Abbreviation	Expanded Term
ADL	Activities of Daily Living
AE	Adverse event
AESI	Adverse events of special interest
ALT	Alanine Aminotransferase
ANCOVA	Analysis of Covariance
aPTT	Activated Partial Thromboplastin Time
AST	Aspartate aminotransferase
BID	Twice daily (from the Latin *"bis in die"*)
BIPAP	Bilevel Positive Airway Pressure
BMI	Body Mass Index
CBP	Childbearing potential
CDC	Centers for Disease Control and Prevention
CFR	Code of Federal Regulations
CI	Confidence interval
COVID-19	Coronavirus Disease 2019
CO_2	Cardon dioxide
CPAP	Continuous Positive Airway Pressure
CRO	Conract Research Organization
CRP	C-reactive protein
CSA	Clinical Study Agreement
DC	Dendritic cell
eCRF	Electronic case report form
EDC	Electronic database capture
eGFR	Estimated glomerular filtration rate
EOI	End of Intake
FDA	Food and Drug Administration
GCP	Good Clinical Practice
GI	Gastrointestinal
GPCR	G-protein Coupled Receptors
GRAS	Generally recognized as safe
HCP	Healthcare Personnel
HDL	High density lipoprotein

HSCT	Hematopoietic Stem Cell Transplantation
ICF	Informed consent form
ICH	International Council for Harmonization
INR	International normalized ratio
IRB	Institutional Review Board
ISF	Investigator Site File
KaSK	Kaleido Study Kit
LDL	Low density lipoprotein
MCH	Mean corpuscular hemoglobin
MCH	Mean corpuscular hemoglobin concentration
MDRO	Multi-drug Resistant Organisms
MedDRA	Medical Dictionary for Regulatory Activities
MP	Monitoring Plan
PI	Principal Investigator
PP	Per-Protocol
PT	Prothrombin time
QC	Quality Control
QOL	Quality of Life
RBC	Red blood cell
RDW	Red cell distribution width
RSV	Respiratory Syncytial Virus
SAE	Serious Adverse Event
SAP	Statistical Analysis Plan
SARS-CoV-2	Novel coronavirus, Severe Acute Respiratory Syndrome 2
SCFA	Short-chain Fatty Acid
SD	Standard Deviation
SOA	Schedule of Assessments
SOB	Shortness of Breath
SP	Study product
SSC	Supportive Self Care
TEAE	Treatment-emergent adverse event
WBC	White blood cell

2 INTRODUCTION

2.1 Background

2.1.1 Oligosaccharides and the Gut Microbiome

The gut microbiome is an ecosystem of >10 trillion microbial cells in the intestine and is responsible for functions that human cells cannot carry out independently (Moya 2016). The gut microbiome plays a significant role in human health and disease by affecting nutrient utilization, colonization resistance, development of the immune system, modulation of host metabolism, and other diverse aspects of the host's physiology (Sommer 2013). A person's diet profoundly affects the composition of the gut microbiota, which has a downstream effect on their physiology, immunity and susceptibility to infectious diseases (Kau 2011).

Non- or low-digestible carbohydrates, including oligosaccharides, have been shown to modulate the composition and/or activity of the gut microbiota through influencing its metabolism, thus conferring a beneficial effect on the host (Bindels 2015; Gray 1975). Some dietary oligosaccharides are susceptible to hydrolytic digestion in the stomach and small intestine. The portion of the orally ingested carbohydrate that is not susceptible to hydrolytic digestion reaches the colon where it is available for fermentation by the colonic microbiota. In the colon, there is a vast repertoire of carbohydrate-active enzymes produced by the complex community of resident-commensal bacteria. Fermentation of carbohydrates by gut bacteria results in the production and release of the short-chain fatty acids (SCFAs) acetate, propionate, and butyrate. These SCFAs serve an important role in the maintenance of healthy gut epithelial function.

Non-digestible oligosaccharides have a very well-established safety profile. Several non-digestible carbohydrates are produced using starting materials composed of glucose monomers and are generally recognized as safe (GRAS), or approved as food additives for use in a variety of foods (GRN 436; GRN 610; GRN 711; 21 Code of Federal Regulations [CFR] 172.841), including infant formula (GRN 233).

A considerable body of information for this group of non-digestible glucose-based carbohydrates has been generated in *in vivo* and *in vitro* toxicology studies (Yoshikawa 2013; Burdock 1999; Wils 2008; Bito 2016) and in human clinical studies including many that were reviewed in the Food and Drug Administration's (FDA) scientific review of isolated and synthetic non-digestible carbohydrates published in November 2016 (FDA 2016). Data from animal toxicology studies of these carbohydrates indicate that the effects observed were expected osmotic and fermentative effects with subsequent changes in the gastrointestinal (GI) tract; there was no evidence for direct target organ toxicity, and the effects appear independent of the oligosaccharide

composition. Further, adverse effects in human clinical studies are limited to GI adverse effects that are easily managed by decreasing the dose amount (Grabitske 2009).

2.1.2 COVID-19

In December 2019, an outbreak of pneumonia of unknown cause presented. By early January 2020, scientists had isolated a novel coronavirus, severe acute respiratory syndrome coronavirus 2 (SARS-CoV-2; previously known as 2019-nCoV), from these patients with virus-infected pneumonia. In February 2020, this severe acute respiratory syndrome was designated as COVID-19 and has since been characterized as a pandemic by the World Health Organization.

While the complete clinical picture with regards to COVID-19 is not fully known, the clinical spectrum of SARS-CoV-2 infection appears to be wide, encompassing asymptomatic infection, mild upper respiratory tract illness, severe respiratory failure and even death.

As of 29 May 2020, the coronavirus COVID-19 has affected 188 countries and territories around the world and two international conveyances. It is estimated that COVID-19 has infected over 4 million people worldwide and has been responsible for over 300,000 deaths. In the U.S., COVID-19 has affected over 1 million people and has been responsible for over 80,000 deaths making it a major cause of mortality and morbidity (Johns Hopkins Coronavirus Resource Center, 2020).

2.2 Study Rationale

There are few randomized controlled trials for patients with COVID-19 in an outpatient setting aimed at assessing physiologic effects of COVID-19, the effects on the gut microbiome structure and function, and feasibility of conducting outpatient studies in this population. Existing studies to date have focused on improving outcomes in patients who have been hospitalized and/or are in Intensive Care Units.

Kaleido Biosciences, Inc. (Kaleido, Sponsor) has developed technology to produce novel, defined mixtures of oligosaccharides synthesized from food-based monosaccharide sources. These glycans belong to a class of oligosaccharide substances which are accepted as safe by regulators worldwide for use in food. KB109 has been determined to be GRAS for the intended use. FDA regulations recognize self-determination of GRAS status, and KB109 is being investigated in this study under the regulations supporting research with food.

Kaleido's products are orally administered, non-absorbed synthetic proprietary oligosaccharide mixtures that modulate the composition and metabolic output of the gut microbiome. These

oligosaccharide mixtures escape digestion by the limited number of human carbohydrate-modifying enzymes in the small intestine and pass into the large intestine where they encounter a vast repertoire of carbohydrate-active enzymes produced by a complex community of resident commensal bacteria. The major products of carbohydrate/oligosaccharide fermentation by gut bacteria are butyric acid, propionic acid, and acetic acid (short-chain fatty acids, SCFAs). These SCFAs serve an important role in the maintenance of healthy gut epithelial function (Arpaia 2013, Smith 2013). Butyrate is used as the primary source of energy for gut epithelial cells and promotes maintenance of epithelial integrity. Maintenance of epithelial integrity is important to prevent inappropriate activation of innate and adaptive immune cells. Butyric acid activates G-protein coupled receptors (GPCRs) displayed on the surface of epithelial and immune cells, as well as inhibits histone deacetylases in the nucleus of these cell types. Propionic acid can also promote gut immune homeostasis by activating these same GPCRs. In addition to producing SCFAs, carbohydrate fermentation results in the growth of commensal bacteria and creates a nutritionally competitive ecosystem in the gut which may restrict the growth of unwanted pathobiont species through multiple mechanisms including acidification of the colon.

Current understanding of the modulation of respiratory virus infectivity by the commensal microbiota of the host and the underlying mechanisms in this regulation are poorly characterized. However, there are substantial data supporting a key role for both gut-derived metabolites (e.g., SCFAs) and the direct interaction and migration of immune cells from gut to lung by the common mucosal immune system in pulmonary infections.

Kaleido's *ex vivo* assay has been used to induce and measure the fermentation of different oligosaccharide mixtures, including KB109, by human fecal samples. SCFAs produced through fermentation can be quantified using gas chromatography-mass spectroscopy analysis. The *ex vivo* assay is performed under anaerobic conditions using media that support the growth of commensal bacteria. In *ex vivo* assays testing KB109, it increased the amount of SCFAs produced over water control by ca. three-fold across multiple fecal communities from healthy donors (Figure 1).

Figure 1: Representative KB109 batch SCFA production average across three healthy fecal communities normalized to water control

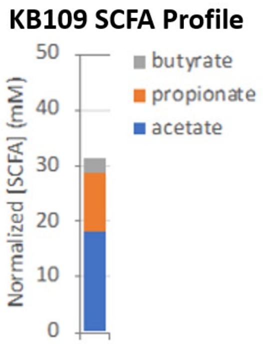

SCFAs modulate host inflammation, control adaptive immunity, and promote immune tolerance locally in the gut as well as systemically (Thaiss 2016; Schirmer 2016). In particular, SCFAs and SCFA-producing taxa have been linked to reduced risk of acquiring viral infections, including corona-viral infections, in at-risk populations such as HSCT patients (Haak 2018). Studies in mice have demonstrated that SCFAs and SCFA-producing microbial taxa, induce virus-specific CD4+ and CD8+ T cells, type 1 interferon, and antibody responses involved in the reduction of viral infection severity (Ichinohe 2011; Trompette 2018). Data has also shown that protection against RSV infection can be conferred by acetate, a metabolite derived from the intestinal microbiota, through induction of IFN-β in the lung through GPR43 and IFNAR-mediated pathways (Antunes 2019). In addition, SCFAs have also been reported to influence macrophage functionality to mitigate neutrophil-mediated tissue damage (Trompette 2018). This is of particular importance as viral infections may be accompanied by an aggressive pro-inflammatory response (Haak 2018) that can elicit a syndrome known as the “cytokine storm”.

In addition to gut microbiome derived metabolites influencing peripheral inflammatory responses, direct activation of host immune cells and pathways by microbiota in the gut has been shown to impact the progression of pulmonary infections. Following influenza virus infection, inflammasome activation can led to migration of dendritic cells (DCs) from the lung to the draining lymph node and T-cell priming. This reveals the importance of commensal microbiota in regulating immunity in the respiratory mucosa through the proper activation of inflammasomes (Ichinohe 2011). Rosshart et al. (2019) reported that reconstitution of the gut microbiota from wild mice confers potent protective effects to laboratory germ free mice during lethal influenza virus infections, an effect mainly mediated through the prevention of an excessive inflammatory response via IL-10 and IL-13. These studies and others suggest a dysbiosis in the microbiome community exhibited as a loss in overall commensal diversity or pathobiont overgrowth might contribute to unfavorable outcomes in respiratory infections.

There is potential that subjects in this study may suffer secondary infections. Kaleido's *ex vivo* screening platform has shown that the KB109 may reduce the relative abundance of multi-drug resistant organisms (MDRO) that colonize the gut, including carbapenem-resistant Enterobacteriaceae (CRE), Vancomycin-resistant Enterococcus (VRE) and C. difficile. The clinical pathogen strains used were obtained from the Centers for Disease Control and Prevention (CDC) and collaborating laboratories and represent multiple sequence types and geographic regions. The experiments described below used fecal samples obtained from ICU patients, as these patients were heavily treated with IV antibiotics and represent the most dysbiotic microbiomes Kaleido has observed to date (Figure 2). In these experiments, the microbial culture obtained upon fermentation of KB109 underwent community composition analysis using shotgun metagenomic sequencing of the entire community genomic DNA. Comparison of the community composition obtained from incubation with or without KB109 allowed measurement of pathogen reduction. To ensure a robust response, each starting community received a CRE or VRE pathogen spike-in (frequently >70% relative abundance of the total community). Note that fecal samples from patient #1, #6, and #12 had virtually no commensal organisms, which we hypothesize explains the lack of Enterobacteriaceae reduction in these samples. Moreover, single-strain experiments demonstrate KB109 does not support the growth of MDR pathogens directly, as measured by optical density (OD600) of the culture (Figure 3). The carbohydrate monomer glucose served as a positive control and supported robust growth of all pathogens. These data further de-risked the ability of KB109 to inadvertently grow gut-colonizing pathogens. By reducing or completely removing the amount of pathogenic organisms that colonize the gut, in addition to improving gut barrier function through the action of microbiome-produced SCFAs, the chance of secondary infection arising from translocation of gut colonizing pathogens is hypothesized to be greatly reduced.

Figure 2: Reduction of Enterobacteriaceae in ICU patients' microbiota (spiked with CRE E.coli) incubated with KB109 relative to water control

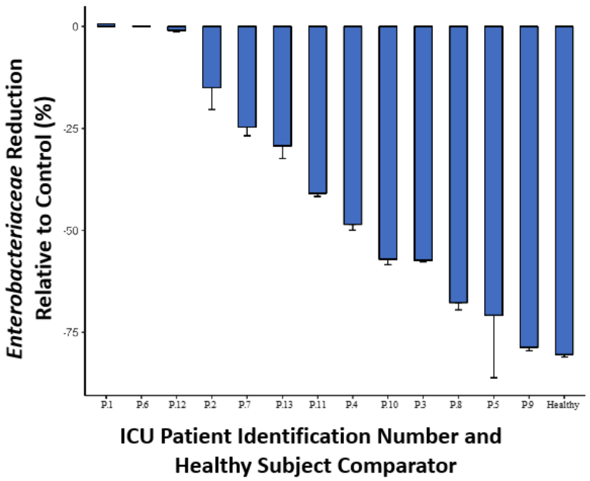

Figure 3: CRE and VRE growth on KB109 demonstrated similar growth to water control, suggesting an inability of these pathogens to directly utilize KB109

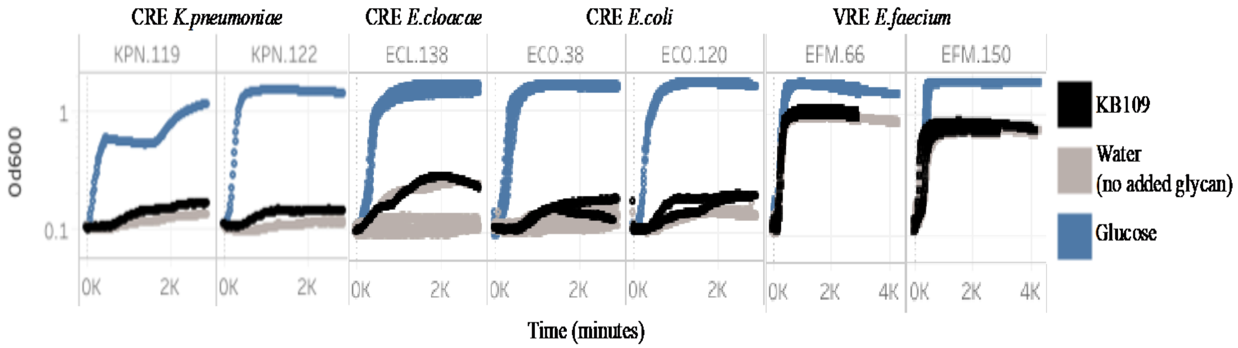

Complications of COVID-19 are known to occur in the patient population eligible for this study, and patients will be monitored for such complications throughout the study. In summary, this is a study designed to evaluate the safety of KB109 and the influences of the microbiome in patients with mild to moderate symptoms of COVID-19 in the outpatient setting. Additionally, given the novel outpatient design, in an emerging disease, this study will also enable assessment of various feasibility indices to inform the design and operations of future clinical trials in COVID-19.

2.3 Benefit/Risk Assessment

KB109 is made from food-based monosaccharide sources, and it belongs to a class of commercially available compounds (non-digestible glucose-based carbohydrates) that have a long history of safe use in food. The Sponsor has engaged an external scientific consulting organization that is frequently used by the food industry to prepare and make GRAS determinations. KB109 was concluded to be Generally Recognized as Safe (GRAS) for use in this study by experts recognized in the field to evaluate GRAS in accordance with the scientific requirements outlined in 21 CFR §170.30(b). This report is a sufficient basis for establishing the GRAS status of KB109 as intended for use as food in this study. FDA regulations recognize self-determination of GRAS status. Although tolerability varies among individuals and among nondigestible carbohydrates, the adverse effects of nondigestible carbohydrates are not qualitatively different from each other and are dose-dependent, quickly reversible, and localized to the GI tract (Grabistke and Slavin 2009).

Kaleido's orally administered proprietary oligosaccharide mixture, KB109, holds promise in inducing a beneficial profile of SCFA production in the gut while driving the growth of commensal bacteria. Increased SCFA and the modulation in microbiome taxonomy may lead to a more appropriate immune and inflammatory response and may prevent an over-aggressive response, up to and including "cytokine storm" observed in patients with COVID-19. KB109 is determined to be GRAS for its intended use in this study and is not intended to treat any condition as it is a food product.

3 STUDY OBJECTIVES AND ENDPOINTS

3.1 Study Objectives

The objectives of this study are to evaluate the following:

- The safety of KB109 in outpatients with mild-to-moderate COVID-19
- The gut microbiota structure in outpatients with mild-to-moderate COVID-19 in the presence and absence of KB109
- Measures of gut microbiome function in outpatients with mild-to-moderate COVID-19 in the presence and absence of KB109
- Measures of health in outpatients with mild-to-moderate COVID-19
- To evaluate the changes in laboratory measures, specific biomarkers, serology and viral load in outpatients with mild-to-moderate COVID-19 in the presence and absence of KB109.
- To evaluate clinical trial feasibility indices

3.2 Study Endpoints

The study endpoints are as follows:

- Number of patients experiencing study product (SP)-related treatment-emergent adverse events (TEAEs)
- **G**ut microbiota structure (e.g., magnitude of change in gut microbiome structure, composition of gut microbiome) by methods such as nucleic acid sequencing prior to Day 1, and at Day 14 (End of Intake, EOI) and Day 35
- **L**evels of stool inflammatory biomarkers (e.g., lipocalin) and gut microbiome metabolites (e.g., SCFA) prior to Day 1, and on Day 14 (EOI) and Day 35
- Measures of Quality of Life (QOL)
- Measures collected from the Healthcare Provider Wellness Visits
- Proportion of patients experiencing hospital admissions during the Intake Period and Follow-up Period
- Healthcare Utilization during the Intake Period and Follow-up Period
- Proportion of patients with oxygen saturation <95%
- Proportion of patients with oxygen saturation <98%
- Proportion of patients with temperature below 100.4 °F without an anti-pyretic medication
- Change from Baseline (Day 1) in:
    - Laboratory measures
    - Biomarkers of infection, serology, and inflammation (e.g., D-dimer, lipocalin, cytokines, IgM/IgG sero-conversion, and Neutralization Assays)
- Aspects of clinical design and conduct including patient agreement with inclusion and exclusion criteria, acceptance of study-specific procedures, technical aspects of trial conduct and data capture, site resource allocation, recruitment strategies, and subject retention

4 STUDY DESIGN

4.1 Overall Design

This study is a randomized, controlled, multi-site, open label clinical study. It is intended to assess KB109 on safety and physiologic effects, effects on the gut microbiome structure and function, healthcare utilization (including hospitalizations), laboratory/biochemical indices and

quality-of-life measures in outpatients who have tested positive with COVID-19, have mild-to-moderate disease, and have been advised to manage their disease at home with SSC under quarantine protocols set forth by the CDC or local ordinances or practices as advised by their healthcare provider (https://www.cdc.gov/coronavirus/2019-ncov/if-you-are-sick/steps-when-sick.html). The study will also collect information regarding feasibility of conducting a study of patients with COVID-19 in an outpatient setting.

Approximately 50 patients will be randomized (1:1) to receive either SSC + KB109 or remain on SSC alone. The randomization will be stratified by site/center, age subgroup (≥18 to <45 years, ≥45 to <65 years, ≥65 years) and comorbidity status (Yes, No). The study consists of a Screening/Randomization Visit, Intake Period (14 Days) followed by a 21-day Follow-up Period (Figure 4).

Details of the procedures and assessments conducted during the study are provided in Section 4.3, and the timing for each is presented in the Schedule of Assessments (SOA; **Table 1**) and summarized in Figure 4.

To be eligible for the study, patients must be at least 18 years of age, have tested positive for COVID-19, and medically stable at study entry per eligibility criteria. Patients must have also been advised by a healthcare provider that self-management of COVID-19 is indicated; eligibility criteria are detailed in Section 5.1and Section 5.2.

Patients will be recruited via outpatient clinics performing SARS-CoV-2 testing and identified by their treating provider as a potential study candidate. Patients will have an opportunity to voluntarily consent into the study at the outpatient testing center/clinic itself or following discharge from the outpatient clinic. Consenting for the study may take place by phone per institutional policies. SARS-CoV-2 test results from a local testing clinic that is not the study site location may be used to determine eligibility; hospital, academic, or industry-based assays will be acceptable for the diagnosis of COVID-19. Home-based tests are not accepted for this study. Eligible patients must be randomized within 48 hours of receiving a positive test result for COVID-19. All enrolled patients will have same level of care and same procedures conducted.

Part 1

This study comprises two parts. In Part 1, patients who consent to the study will undergo a full assessment of inclusion and exclusion criteria, have blood samples collected for Baseline hematology, chemistry, biomarkers and serological markers of immunity, and may have nasal and oropharyngeal swabs taken for quantitative vial load assessments (See **Table 1** and

eligibility criteria in Section 5.1, and Section 5.2). Patients will be asked to sign into a secure website called “TrialPace™ diary” (TrialPace) with a unique log-in and password.

It is possible that patients may only consent to Part 1 of this study initially. In this case, if a positive test result for COVID-19 is received, then the patient may be contacted by the study team (or designee such as a centralized telemedicine provider, physician or nurse practitioner) to review study eligibility criteria, obtain informed consent to Part 2 per institutional policies (e.g., remote consenting or consenting by phone per institutional policies). The patient may also choose to provide consent to both Part 1 and Part 2 at the initial intake.

Information entered into TrialPace for patients who are deemed ineligible for the study will not be used for any purpose.

Part 2

Only patients who have a positive test result for COVID-19 can continue to Part 2 of this study. Part 2 of the study comprises an Intake Period and a Follow-up Period. In Part 2, following confirmation of eligibility criteria, the site will notify an independent, third party vendor that a patient is eligible for randomization no later than 48 hours after a positive test result for COVID-19. Upon randomization (SSC + KB109 or SSC alone), a Kaleido at-home Study Kit (KaSK) and SP (KB109) and dosing instructions (as applicable) will be shipped via overnight delivery to the patient’s home. The KaSK will include a thermometer, and a pulse oximeter. Patients will also be provided with the telemedicine contact information

Part 2, Intake Period

The Intake Period (Days 1-14) will begin the morning after receipt of the KaSK. All patients will continue to follow the SSC guidance as provided by the treating healthcare provider throughout study participation. In the Intake Period, all patients will be asked questions related to quality of life measures, healthcare utilization measures, and concomitant medications taken in the previous 24 hours and will record responses in TrialPace where appropriate. Patients randomized to SSC + KB109 will begin consuming KB109 on Day 1 after recording the required information. All KB109 usage will be noted in the patient’s SP log. For all patients in both groups, temperature and oxygen saturation will be measured and recorded in TrialPace as needed throughout the day prior to taking anti-pyretic medication. Additionally, patients in both groups will be asked to collect a stool sample prior to the first study product intake for patients in the KB109 + SSC group or prior to the morning of Day 1 for the patients in the SSC Alone group.

During the Intake Period, for all patients, blood, nasal and oropharyngeal swabs may be collected in the clinic, at home by a healthcare professional or remotely, as feasible, and as close to the beginning (Day 1) and end (Day 14) of the Intake Period for analysis of laboratory, biochemical and serological measures. Patients will have wellness visits by telephone on Days 3, 7, 10 and 14 to follow-up on the patient's health status and to ascertain compliance with SP usage or completion of TrialPace questions, and to re-education the patient on the importance of adherence to study instructions including SP usage (where applicable).

On Day 14, all patients will undergo another telemedicine visit where an abbreviated physical examination will be conducted, an assessment of safety, and an evaluation of whether follow-up treatment is recommended due to a deterioration of COVID-19 symptoms. When wellness and telemedicine visits fall on the same day, they will be combined as a telemedicine visit. Patients in the SSC + KB109 group will stop taking KB109 on Day 14. Additionally, patients in both groups will be asked to collect a stool sample within 2 days of or on Day 14 (i.e., Days 12-14).

Part 2, Follow-up Period

On Day 15, all patients will enter the Follow-up Period (Days 15 to 35) where physiologic measurements (temperature and oxygen saturation), quality of life and healthcare utilization indices will be collected weekly and patients will record responses in TrialPace where appropriate. Wellness visits by telephone will be conducted on Days 21 and 28. Blood, nasal and oropharyngeal swabs for analysis of laboratory, biochemical and serological measures may be collected at home by a healthcare professional or remotely, as feasible, and as close to the end of the Follow-up Period (Day 35). Additionally, patients in both groups will be asked to collect a stool sample within 2 days of or on Day 35 (i.e., Days 33-35).

On Day 35, all patients will undergo another telemedicine visit (including wellness assessments) where an abbreviated physical examination will be conducted, an assessment of safety, and an evaluation of whether follow-up treatment is recommended.

For convenience, standard terms language included in International Conference for Harmonization (ICH) guidance relevant to the conduct of clinical trials are used in this protocol. An example of such a term used in this protocol is "treatment emergent," included in ICH E9. Use of this standard term is not intended to connote treatment with a medicinal product administered (ICH E9 2018).

Throughout the study, safety will be monitored by TEAEs, clinical signs and symptoms, and selected vital signs (i.e., temperature and oxygen saturation). Concomitant medications/supplements will be recorded throughout the study. Any patient experiencing a TEAE, or intolerable GI symptoms will be evaluated by the principal investigator (PI) or telemedicine provider for possible interruption and/or down-titration of SP dose to the previously tolerated level in consultation with the medical monitor.

In the event of significant worsening of COVID-related symptoms or two sequential pulse oximetry readings of <94% within 2 minutes, patients will be instructed to seek medical attention immediately (See Section 6.7).

In the event of worsening GI symptoms of other attributable AEs, the SP may be down titrated or temporarily interrupted (See Section 6.8). Details of the patient's SP dose (including the date, time, and amount) will be recorded in SP dosing logs.

See Section 4.3 for further information on the study procedures, and Table 1 for the Schedule of Assessments.

Figure 4: Study Design

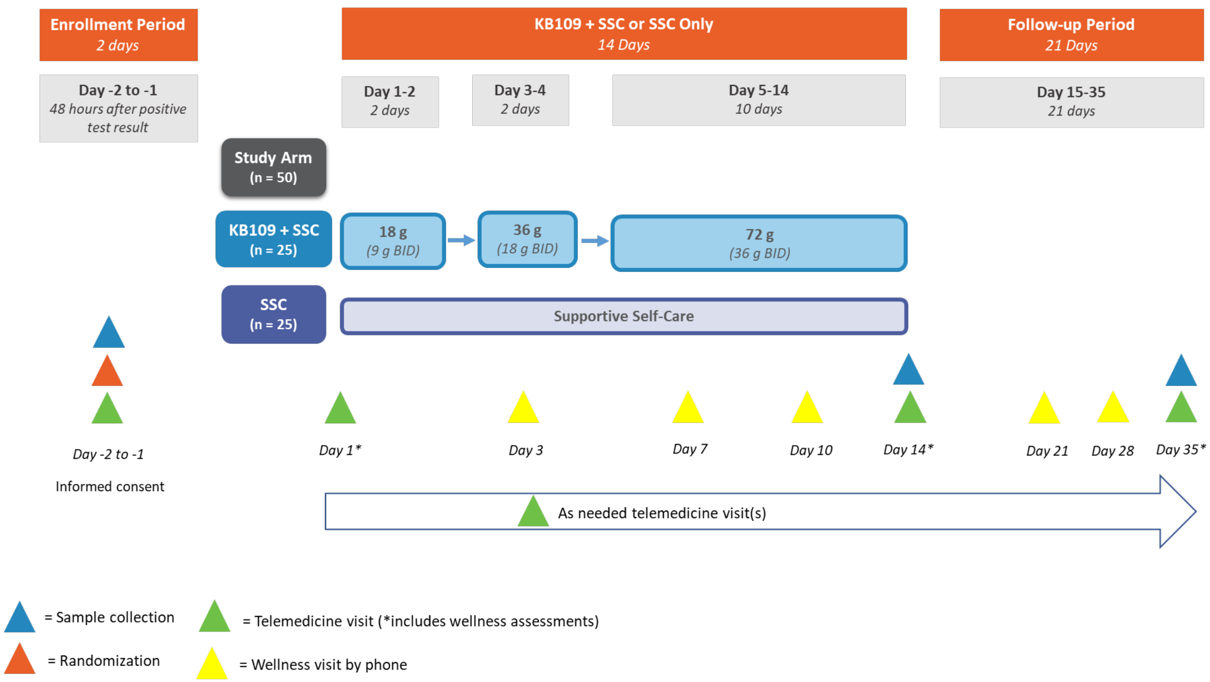

4.2 Study Duration

The total duration of the study per study patient is approximately 37 Days (Screening/Randomization [Day -2] through to the end of the Follow-up Period [Day 35]), (see Figure 4).

4.3 Study Procedures

Details of the individual study procedures are provided in Section 4.3.1 through Section 1.1.1. Timing for when each procedure is to be conducted is presented in the SOA below (Table **1**). All self-assessments and patient-reported responses will be recorded in TrialPace using the patient's unique log-in and password.

Table 1. Schedule of Assessments

Assessment	Part 1	Part 2										
	Screening	Intake Period: SSC + KB109 or SSC Only						Follow-up Period				Early Termination Visit
	Up to Day -2 to -1	Day 1	Day 2	Day 3	Day 7	Day 10	Day 14 ± 3 days	Day 15 to Day 20	Day 21	Day 28	Day 35	
Informed Consent	X											
Registration in TrialPace	X											
Demographics and medical history	X											
Confirm eligibility criteria[1]	X											
Randomization[2]	X											
Consume KB109 for subjects in this group (SSC + KB109)		X	Daily				X					
Physical Examination	X	X[3]					X[3]				X[3]	X
Height, weight, BMI calculation	X											
Patient-assessed COVID-19 Signs (temperature and oxygen saturation)[4]	X	X[5]	Daily				X		X	X	X	X
Quality of life indices							X				X	
Healthcare utilization questions[4]			Daily				X	Daily				X
Nasal swab[5]	X						X				X	
Oropharyngeal swab[5]	X						X				X	
Blood samples[5]	X						X				X	
Stool samples[6]		X					X				X	X
Telemedicine Visit[7]		X					X				X	
Wellness Visit by Telephone[8]		X		X	X	X	X		X	X	X	
AE review	Throughout the study											
Concomitant medication	Throughout the study											
Return of study product and other materials[9]											X	X

[1]Note that the patient will have had a SARS-CoV-2 test at their outpatient clinic. This test is not part of the study procedures. Additionally, pregnancy status will be collected by patient self-report; if there is any uncertainty about pregnancy status, patients may be provided with a pregnancy test kit (urine test approved for home use) and results will be reported by the patient to a healthcare provider on the study team to confirm eligibility.

[2]Eligible patients must be randomized within 48 hours of positive SARS-CoV-2 test result.

[3]Physical examination at the telemedicine visit will include a general assessment (e.g., overall appearance, orientation) and an assessment for work of breathing (e.g., speech cadence, lifting hands above head).

[4]The Patient-assessed COVID-19 signs (temperature, oxygen saturation) and Healthcare utilization responses are recorded in TrialPace. On Day 1, these measures should be completed prior to the first dose of KB109 for patients in the SSC + KB109 group.

[5]See Section 4.3.7 for the blood tests. The first blood samples will be taken at the clinic at the time of testing for COVID-19 or within 48 hours of randomization. After randomization, blood samples can be drawn on Days 13-15 during the Intake Period and Days 33-35 during the Follow-up Period. If feasible, nasal and oropharyngeal swabs may be taken at the same times as blood samples.

[6]Stool samples should be collected prior to the first study product intake on Day 1 for participants in the KB109 + SSC group and prior to the morning of Day 1 for participants in the SSC alone group. Stool samples for both groups are also collected within 2 days of or on Day 14 (i.e., Days 12-14) and Day 35 (i.e., Days 33-35).

[7]Telemedicine Visits will take place on Days 1 (+ 1 day), 14 (±3 days), and 35 (±1 day), and will include Wellness visit assessments on these days. Telemedicine or Wellness visits may also take place throughout the study as needed.

[8]Wellness assessments will be included in the Telemedicine visits when they fall on the same day. A visit window of ±1 day is allowed for Wellness visits.

[9]Study product reconciliation may take place virtually.

4.3.1 Patient Demographics and Baseline Characteristics

Demographic information and baseline characteristics obtained will include age, sex, race, ethnicity, weight, and height.

4.3.2 Medical History and Concomitant Medications/Procedures

The medical history of the patient will be obtained by the PI or qualified designee. Medical history will be recorded and should include prior/existing medical conditions and surgical procedures, physical examination findings and clinically significant laboratory abnormalities.

Specific detailed information regarding the reason for COVID-19 testing, clinical symptoms, complications, and etiology of COVID-19 will also be collected. Ongoing conditions are considered concurrent (comorbid) medical conditions.

Medication history information to be obtained also includes any medication (prescription or nonprescription) and any dietary or nutritional supplement relevant to eligibility criteria stopped at or within 28 days before signing of informed consent.

Concomitant medications and procedures will be recorded throughout the study.

4.3.3 Physical Examination

In order to minimize contact between patients and providers, a basic face-to-face physical examination performed by a qualified healthcare provider in accordance hospital policies and procedures at the patient's outpatient clinic visit will be used for assessing study eligibility at the Screening visit. The basic face-to-face physical examination will include general assessment (e.g., overall appearance, orientation), pulmonary auscultation, and an assessment for work of breathing (e.g., speech cadence, lifting hands above head, observing ambulation).

Abbreviated telemedicine-based physical examinations include a general assessment (e.g., overall appearance, orientation) and an assessment for work of breathing (e.g., speech cadence, lifting hands above head), and will be performed according to guidelines outlined via the central telemedicine provider at the telemedicine visits and as needed throughout the study (see **Table 1**). All findings from this examination will be recorded on source documents and entered into the eCRF.

Any new abnormal findings upon physical examination noted after informed consent is obtained and deemed clinically meaningful by the PI or designee should be recorded in the eCRF as an AE.

4.3.4 Patient-assessed COVID-19 Signs

Following randomization, selected patient-assessed COVID-19 signs will be performed daily, upon wakening, as needed and according to the SOA (Table **1**).

Patients will self-assess temperature and oxygen saturation according to the instructions for use of the thermometer and pulse oximeter included in their KaSK. See Section 6.7 regarding patient instructions in the event COVID-19 signs and symptoms worsen.

4.3.5 Quality of Life Indices

Following randomization, on Days 14, and Day 35, during the telemedicine visit, the telemedicine provider will ask the following questions to assess quality of life (Table **1**). Telemedicine providers must read the questions as they are written and cannot provide the patient with assistance on interpreting the meaning of questions as it is paramount that the interpretation be left up to the patient.

1.) Overall, how would you rate your health during the past 14 (or 21) Days?

Rated as: Excellent, Very Good, Good, Fair, Poor, Very Poor

2.) During the past 14 (or 21) Days, how much did your COVID-19 symptoms limit your physical activities (such as walking or climbing stairs)?

Rated as: Not at all, Very little, Somewhat, Quite a lot, Could not do physical activities

3.) How much bodily pain have you had during the past 14 (or 21) Days?

Rated as: None Very mild, Mild, Moderate, Severe, Very severe

4.) During the past 14 (or 21) Days, how much energy did you have?

Rated as: Very much, Quite a lot, Some, A little, None

5.) During the past 14 (or 21) Days, how much have you been bothered by emotional problems (such as feeling anxious, depressed or irritable)?

Rated as: Not at all, Slightly, Moderately, Quite a lot, Extremely

6.) Thinking about any emotional problems you may have had such as feeling, anxious, depressed or irritable, for how many days during the past 14 (or 21) Days was your emotional health not good?

Rated as number of days from 1-14 days at the end of the Intake Period (Day 14) and 15-35 at the end of the Follow-up Period.

7.) Thinking about your overall physical health for how many days during the past 14 (or 21) Days was your physical health not good?

Rated as number of days from 1-14 days at the end of Intake Period (Day 14) and 15-35 at the end of the Follow-up Period.

8.) During the past 14 (or 21) Days, how much did your COVID-19 symptoms interfere with your normal sleep patterns?

Rated as: Not at all, Slightly, Moderately, Quite a lot, Extremely

4.3.6 Healthcare Utilization

Following randomization, patient-assessed measures of healthcare utilization will be performed every morning upon wakening and according to the SOA over 35 days (Intake Period plus Follow-up Period) (**Table 1**).

Measures will include:

- Prescription Medication Use – recorded as reason, type, dose and frequency
- Over-the-counter Medication Use – recorded as reason, type, dose and frequency
- Supplement Use – recorded as reason, type, dose and frequency
- Emergency room or Urgent Care visit – Rated as Y/N; if Y then reason will be recorded
- Medical provider visit – Rated as Y/N; if Y then reason as well as if this is a visit for a co-existing condition (mental health visit, physical therapy, etc) will be recorded;
- Hospital stay – Rated as Y/N; if Y then reason will be recorded

4.3.7 Laboratory and Inflammatory Biomarkers

Blood samples will be collected for hematology, chemistry, serology and quantitative vial load assessments throughout the study. If feasible, nasal and oropharyngeal swabs may be collected for quantitative viral load (for research purposes only). Also refer to the SOA (**Table 1**). Samples will be collected in accordance with acceptable laboratory procedures (**Table 2**); additional analyte values may also be reported, consistent with the study endpoints and objectives.

Table 2. Clinical Laboratory Tests

Hematology	Chemistry		Nasal and Oropyarngeal Swabs
RBC<br>RDW<br>MCH<br>MCHC<br>WBC with differential (% and absolute)<br>Hemoglobin<br>Hematocrit<br>Platelets<br>aPTT<br>PT<br>INR	ALT<br>Albumin<br>Alkaline phosphatase<br>AST<br>Total bilirubin<br>Total protein<br>Creatinine<br>Blood urea nitrogen<br>Magnesium<br>Phosphorus<br>CRP<br>Ferritin<br>D-dimer	Potassium<br>Sodium<br>Glucose<br>Chloride<br>Bicarbonate or CO_2<br>Calcium<br>eGFR<br>Total cholesterol<br>HDL cholesterol<br>LDL cholesterol<br>Triglycerides	Quantitative Viral Load (not for diagnostic purposes)

Abbreviations: aPTT: activated partial thromboplastin time, ALT: alanine aminotransferase, aPTT: activated partial thromboplastin time, AST: aspartate aminotransferase, CO_2: carbon dioxide, CRP: C-reactive protein, eGFR: estimated glomerular filtration rate, HDL: high density lipoprotein, INR: international normalized ratio, LDL: low density lipoprotein, MCH: mean corpuscular hemoglobin, MCHC: mean corpuscular hemoglobin concentration, PT: prothrombin time, RBC: red blood cell, RDW: red cell distribution width, WBC: white blood cell.

Note: the list of laboratory tests may not be inclusive of all tests conducted throughout the study.

Blood samples may also be assessed for markers of infection, antibody response, and inflammation such as:

- D-dimer, lipocalin, cytokines, IgM/IgG sero-conversion, and Neutralization Assays

Instructions relating to blood and nasal and oropharyngeal swab collection methodologies storage requirements, and shipment requirements will be detailed in the Laboratory Manual.

4.3.7.1 Stool Sample Collection

Instructions for collecting, and specific methodology for stool handling and/or storage of stool samples, will be provided in a separate Laboratory Manual. Participants will be provided a stool

collection kit and instructions to collect these samples in their home. Samples should be stored immediately per the instructions in the Laboratory Manual and at-home instructions. Samples may be shipped to the central laboratory via courier or pickup/shipment by a third-party healthcare professional contracted by the Sponsor. Upon delivery of stool samples to the central laboratory or sample storage location, samples will be stored immediately at -75°C ± 5°C until analysis.

Microbiome Structure and Composition
To assess differences in the gut microbiome in the SSC + KB109 compared to the SSC alone group, nucleic acid sequencing will be used to determine alpha diversity, taxonomy and abundance of bacteria in stool.

Biomarker Analysis in Stool
Inflammatory biomarkers, such as calprotectin, and lipocalin and others, and gut microbiome metabolites (e.g., SCFA) may be evaluated in fecal samples. Note: This is not an all-inclusive list of biomarkers and metabolites. Additional biomarkers and metabolites may be considered in the future as other candidates increase in importance.

4.3.8 Telemedicine Visit

As outlined in the SOA (**Table 1**), a healthcare provider from a centralized telemedicine vendor will conduct a telemedicine study visit. This visit may include a physical examination and may be used to interview and collect safety information along with other protocol-specified measures of health as needed.

4.3.9 Wellness Visit by Telephone

As outlined in the SOA (**Table 1**), a healthcare provider from a centralized telemedicine vendor will conduct a wellness visit by telephone. This phone call to the subject will be conducted to follow-up on the subject’s health status and to ascertain compliance with study procedures.

5 STUDY POPULATION

5.1 Inclusion Criteria

To be considered for enrollment into this study, each patient must meet **all** of the following Inclusion Criteria:

1. Adult ≥18 years of age
2. Be willing and able to give informed consent, and provide medical history, and secondary contact information
3. Screening/Randomization within 2 days of testing positive test for COVID-19
4. Mild-to-moderate COVID-19 as having self-reported fever, or cough (new or exacerbated chronic) or presence of at least 2 of the following: anosmia, sore throat, or nasal congestion for not more than 72 hours prior to COVID-19 testing
5. Mild to moderate COVID-19 and self-reported outpatient management indicated by their healthcare provider
6. Able to adhere to the study visit schedule and other protocol requirements
7. Has consistent internet or cellphone access with a data plan, and access to a smartphone, tablet or computer

5.2 Exclusion Criteria

Patients who meet **any** of the following Exclusion Criteria will not be enrolled into the study:

1. In the Primary Investigator's judgement, patients likely to require hospitalization for COVID-19
2. Patients who are hospitalized for in-patient treatment or currently being evaluated for potential hospitalization at the time of informed consent for conditions other than COVID-19
3. History of chronic lung disease with chronic hypoxia
4. History of documented cirrhosis or end-stage liver disease
5. Ongoing requirement for oxygen therapy
6. Shortness of breath in resting position
7. Diagnosis of sleep apnea requiring Bilevel Positive Airway Pressure (BIPAP) / Continuous Positive Airway Pressure (CPAP)
8. Female patients who are pregnant, trying to become pregnant or lactating.
9. Concurrent use of any of the following medications:
    a. Therapy with an immunomodulatory agent within 12 months of study screening
    b. Systemic antibiotics, antifungals, or antivirals for treatment of active infection within 28 days of study screening
    c. Systemic immunosuppressive therapy within 3 months of study screening
    d. Drugs or other compounds that modulate GI motility (including stool softeners, laxatives, or fiber supplements) taken currently, or within 7 days of Study screening
10. History of GI surgery (6 months prior to Randomization), including but not limited to bariatric surgery and bowel resection, or history of, or active GI disease(s) that may affect assessment of tolerability, including but not limited to the following:

    a. Inflammatory bowel disease
    b. Irritable bowel syndrome
    c. Autoimmune disease
    d. GI malignancy
11. Participation in an interventional clinical trial with or use of any investigational agent within 30 days before Randomization
12. Has a clinically significant or uncontrolled concomitant medical condition that would put the patient at risk or jeopardize the objectives of the study in the opinion of the PI
13. Is considered, in the opinion of the PI, to be unlikely for any reason to be able to comply with study procedures
14. Contraindications, sensitivities, or known allergy to the use of the study product or its components

5.3 Screen Failures

Patients who provide informed consent but do not meet the study entry criteria during Screening and consequently are not enrolled in the study are screen failures. Patients may not be re-screened.

5.4 Enrollment

Patients will be randomized in a 1:1 ratio to either SSC + KB109 or SSC alone; see Section 6 for further information on the SP. Consented patients who are withdrawn from the study prior to randomization are deemed screen failures; patients withdrawing after randomization will not be replaced nor re-consented for new participation.

5.5 Withdrawal of Patients

Patients have the right to withdraw from the study at any time for any reason, without giving a reason and without penalty or loss of benefits they are entitled to. The investigator also has the right to withdraw patients from the study if this is in the best interest of the patient.

Patients will be discontinued from the study in the event of any of the following:

- Patient independently withdraws consent from the study (e.g., patient declines further participation in the study)
- PI determines that the patient has developed a concurrent illness, condition, or an AE due to which continued participation in the study and/or SP dosing is considered potentially harmful to the patient and withdrawal is in the best interest of the patient
- Patient discontinues from SP dosing (i.e., due to development of an AE), but continues his/her consent to participate in the study for follow-up only (e.g., for continued collection of AE information)

- Patient failure to comply with study requirements (e.g., failure to properly consume the SP or failure to comply with other protocol-specific assessments) resulting in protocol deviations that make the interpretation of the data not feasible
- Patient becomes pregnant
- Patient requires hospitalization due to worsening COVID-19

5.5.1 Handling of Discontinuation of SP and Early Terminations

If the PI discontinues a patient from SP due to development of an AE, the patient wishes to withdraw from the study, or the patient is terminated from the study for any other reason every effort should be made to:

- Complete the Early Termination visit assessments, as directed in the SOA (**Table 1**)
- Monitor the patient for resolution of any existing AEs, and onset of new AEs for 3 weeks (21 days) after the last dose of SP.

The data should be documented on the appropriate eCRF in the electronic database capture (EDC) system.

If, due to extenuating circumstances, the PI and/or study site team are concerned that the patient may not be able to safely complete all the required visits and assessments the PI should contact the Sponsor’s Medical Monitor to discuss whether an early termination visit should occur to enable the return of the SP, and the completion of key end of intake assessments/measures, whilst ensuring the patient’s safety.

5.5.2 Premature Termination or Suspension of Study

This study may be suspended or prematurely terminated at the Sponsor’s discretion. Written notification documenting the reason for study suspension or termination will be provided by the Sponsor to the PI. If the study is prematurely terminated or suspended, the PI will promptly inform the Institutional Review Board (IRB) and will provide the reason(s) for the termination or suspension.

Circumstances that may warrant termination or suspension include, but are not limited to:

- Determination of unexpected, significant, or unacceptable risk to patients as determined by the ongoing review of aggregate safety data by the medical monitor

Circumstances that may warrant termination or suspension of an individual site include but are not limited to:

- Insufficient compliance with protocol requirements by the site
- Site submits data that are consistently insufficient, incomplete, and/or unevaluable

If the study is suspended temporarily at a site, the study may resume once concerns about safety, protocol compliance, and/or data quality are addressed and satisfy the Sponsor and/or IRB.

5.5.3 Lost to Follow-up

A patient will be considered lost to follow-up if he or she repeatedly cannot be contacted for scheduled telemedicine visits or sample collections and is unable to be contacted by the study site.

If a patient fails to attend a required study visit, the site must:

- Attempt to contact the patient and reschedule the missed visit as soon as possible
- Counsel the patient on the importance of maintaining the assigned visit schedule
- Ascertain whether the patient wishes to and/or should continue in the study

Before a patient is deemed lost to follow-up, the PI or designee must make every effort to regain contact with the patient until the end of the study, including potentially contacting the emergency contact listed by the patient. These contact attempts should be documented in the patient's study record. Should the patient continue to be unreachable, he/she will be considered lost to follow-up.

6 STUDY PRODUCTS

6.1 Description of Study Products

6.1.1 KB109

As depicted in Figure 5, KB109 is a mixture of polymers of glucose, galactose, and mannose in proportions of approximately 45%, 45%, and 10%, by weight respectively. The formula is $H\text{-}[C_6H_{9\text{-}11}O_5]_n\text{-}OH$, where the total number of monomer units in a single polymer of the mixture ranges from 2 to approximately 60 (n = 2-60), with a mean value for the mixture of approximately 12.6 monomer units. Each monomer unit may be unsubstituted, singly, doubly, or triply substituted with another glucose, galactose, or mannose unit by any glycosidic isomer.

Figure 5: KB109 Structure

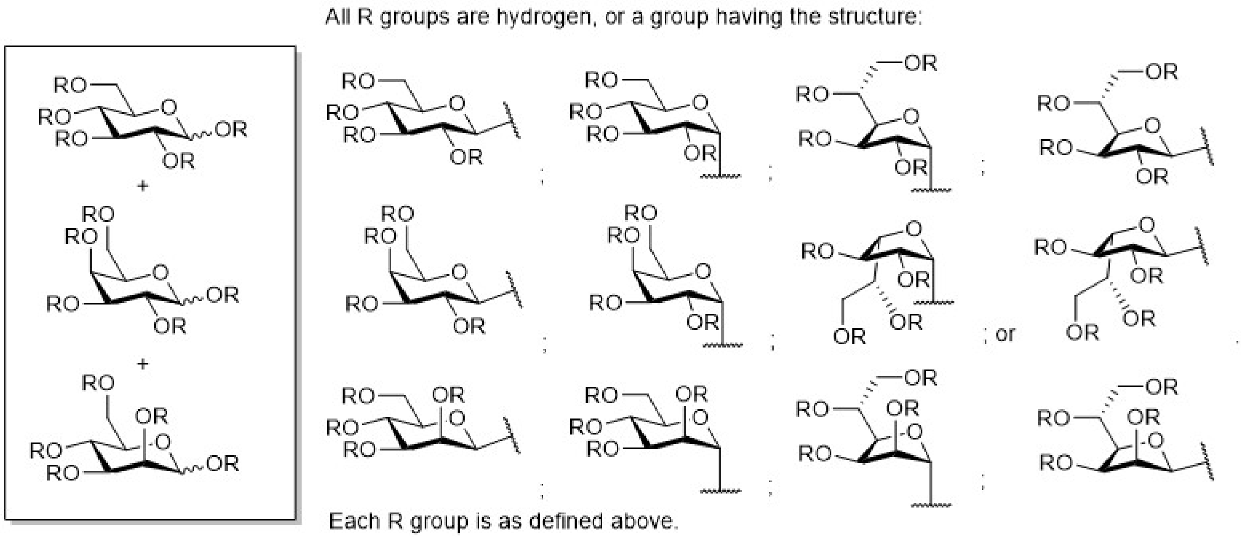

6.2 SP Dosing, Preparation and Administration

Patients randomized to SSC + KB109 will consume their SP orally BID (at least 8 hours apart). Preparation of the SP involves adding 120 mL of water into a glass/container, instilling the directed amount of SP powder on top, and mixing to dissolve prior to drinking as a beverage. The SP should be consumed within 1 hour after reconstitution.

Dosing of KB109 is detailed in **Table 3**.

Table 3. Dose Up-titration of the Study Product

SP Administration Period	Dose (KB109)
Days 1–2	9 g BID (18 g/day)
Days 3–4	18 g BID (36 g/day)
Days 5–14	36 g BID (72 g/day)

BID: Twice daily; SP: Study Product.

Doses should be taken at the same time each day. The morning dose should be taken by noon each day, and the evening dose should be taken approximately 8 hours after the morning dose. A missed morning dose may still be taken before noon on the same day; a missed evening dose may be taken within 4 hours of the routine daily evening administration dose.

Details of the patient's SP dosing (including the date, approximate time, and amount) will be recorded in SP dosing logs.

6.3 SP Storage and Accountability

The SP is to be dispensed by the trained team at the storage and shipping vendor. SP reconciliation may take place virtually. Patients will be instructed to return used and unused SP to the storage and shipping vendor, per the instructions included in the SP packaging. At the termination of the study or at the request of the Sponsor, the storage and shipping vendor will destroy or return unused SP and all partially dispensed or empty packets.

6.4 Unblinding

This is an open label study. There are no requirements for unblinding.

6.5 Concomitant Medications/Dietary Supplements

All concomitant medications and dietary supplements taken after consent is provided will be recorded. Medications to be reported include all prescription medications, over-the-counter medications, and other non-prescription medications taken by a patient during study participation. All dietary supplements taken by patients during study participation will be reported as well. For this study, a prescription medication is defined as a medication that can be prescribed only by a properly authorized/licensed clinician.

Information regarding eligibility criteria relating to the use of certain medications can be found in Section 5.1 and Section 5.2 of the protocol.

See Section 6.6 for prohibited medications and dietary supplements.

6.6 Prohibited Medications/Dietary Supplements

Medications or dietary supplements prohibited at screening are also prohibited during the study provided that appropriate safety considerations are followed, including:

- GI motility agents including stool softeners, laxatives, or fiber supplements (also prohibited within 7 days prior to screening)
- Systemically administered immunosuppressant medications
- Therapy with an immunomodulatory agent within 12 months of study Randomization
- Systemic antibiotics, antifungals, or antivirals for treatment of active infection within 28 days of study Randomization

- Systemic immunosuppressive therapy within 3 months of study Randomization

Additionally, medications that would interfere with the objectives of this study, pose a safety risk, or confound the interpretation of the study results (in the PI's judgement) are prohibited while a patient is participating in the study. This includes oral and systemically acting antibiotics and antifungals.

Should circumstances that require use of prohibited medications arise (such as such as anti-viral or immunomodulating medication), the PI (in consultation with the Medical Monitor and the Sponsor) will make the assessment whether to discontinue the patient from the study for the patient to receive any necessary and appropriate medical care.

6.7 Self-supportive Care

Patients with COVID-19 should follow the steps as instructed by their healthcare provider (consistent with guidance from the CDC, local ordinance or practices) to care for themselves and to help protect other people in the home and community from potentially getting sick (https://www.cdc.gov/coronavirus/2019-ncov/if-you-are-sick/steps-when-sick.html).

In order to manage the symptoms associated with COVID-19, over-the-counter cough, cold, and anti-pyretic medications can be used as necessary by patients in accordance with their respective drug facts label or as instructed by their healthcare provider.

The brand name, generic name, dose, and time must be recorded for every usage occasion.

Since this in an out-patient based study, in the event of significant worsening of COVID-19-related symptoms or two sequential pulse oximetry readings of <94%, patients will be instructed to seek medical attention immediately with their treating provider or go to the emergency room. If the PI or study staff are contacted due to significant clinical worsening of COVID-19 symptoms, the patient will be triaged appropriately to the emergency room or to the outpatient treating provider per PI judgement. The patient will be removed from the study only if hospitalized (refer to Section 5.5).

Patients will be provided with information consistent with CDC guidance regarding emergency warning signs for COVID-19 (https://www.cdc.gov/coronavirus/2019-ncov/if-you-are-sick/steps-when-sick.html) instructing them to seek medical attention immediately, if any of the following occur:

- Trouble breathing

- Persistent pain or pressure in the chest
- New confusion or inability to arouse
- Bluish lips or face

A medical provider must be contacted for any other symptoms that are severe or concerning to the patient. Patients will be instructed to call 911 if they have a medical emergency and be instructed to notify the operator that they have, or think they might have, COVID-19.

Patients will be instructed to put on a cloth face covering before medical help arrives.

6.8 Dose Reductions and Holds

In the event of significant worsening of COVID-19 symptoms, severe or intolerable GI symptoms, or other attributable AEs, the KB109 dose may be down-titrated to a lower dose, or temporarily interrupted, after consultation with the Medical Monitor.

KB109 down-titration occurs by reducing the dose in a step-wise fashion. Specifically, a patient's dose can be reduced after evaluation by the PI or designee, and in consultation with the Sponsor's medical monitor or designee from 72 g/day (36 g BID) to 36 g/day (18 g BID) followed by 18 g/day (9 g BID) until the attributable AE resolves or adequate tolerability is achieved.

During the Intake Period, and at the discretion of the PI and in consultation with the Medical Monitor, patients who have down-titrated their KB109 dose may attempt to increase their dose. This up-titration can only occur at the next up-titration period. Patients can also remain at the down titrated dose, for the remainder of the intake period.

If the patient requires down-titration below 18 g/day (9 g/dose BID), then consultation with the Medical Monitor and PI or designee must occur, and the patient may be withdrawn from the study.

6.9 Other Study Restrictions

Female patients of childbearing potential (CBP) must indicate that they will use one highly effective form of birth control from the day of the first SP dose through the study and for 90 days after the last dose of SP.

6.10 Randomization, Stratification and Blinding

All patients deemed eligible for the study will be randomized in a 1:1 ratio to SSC + KB109 or SSC alone group using an interactive response technology (IRT) system. Randomization will be stratified by study site/center, age group (≥18 to <45 years, ≥45 to <65 years, ≥65 years) and comorbidity status (Yes, No).

The study is an open-label study; therefore, no blinding is necessary.

7 ADVERSE EVENT REPORTING

7.1 Definitions of AEs, Period of Observation and Recording of AEs

For convenience, standard terms language included in ICH guidance relevant to the conduct of clinical trials are used in this protocol. An example of such a term included in this protocol is “treatment-emergent,” included in ICH E9. Use of this standard term is not intended to connote treatment with a medicinal product administered.

An AE is defined as any untoward medical occurrence in a patient involved in a clinical study administered a SP and that does not necessarily have a causal relationship with the product. An AE can, therefore, be any unfavorable and unintended sign (including a clinically significant abnormal laboratory finding which has worsened from baseline), symptom or disease temporally associated with the use of a product, accidents, whether or not considered related to the product or study-related procedure.

All AEs are collected from the time the informed consent is signed until the end of the Follow-up Period. This includes events occurring during the Screening Period of the study, regardless of whether or not SP is administered. Where possible, a diagnosis rather than a list of symptoms should be recorded. If a diagnosis has not been made, then each sign or symptom should be listed individually. All AEs should be captured on the appropriate AE pages in the eCRF and in source documents.

All AEs must be followed to closure (the patient’s health has returned to his/her baseline status or all variables have returned to normal), regardless of whether the patient is still participating in the study. If an AE should extend beyond the subject’s participation in the study, the PI or designee will make every effort to continue to follow the AE until closure. Closure indicates that an outcome is reached, stabilization achieved (the PI does not expect any further improvement or worsening of the event), or the event is otherwise explained. When appropriate, medical tests and examinations are performed so that resolution of event(s) can be documented.

7.2 AE Assessments

7.2.1 Severity

The severity of AEs must be recorded, including the start and stop dates for each change in severity. An event that changes in severity should be captured as a new event. After initiation of KB109, worsening of events that started prior to initiating intake of KB109 must be recorded as new AEs (for example, if a patient experiences mild intermittent dyspepsia prior to dosing of KB109, but the dyspepsia becomes severe and more frequent after first dose of KB109 has been administered, a new AE of severe dyspepsia [with the appropriate date of onset] is recorded in the eCRF).

The medical assessment of severity is determined by using the following definitions:

Mild: A type of AE that is usually transient and may require only minimal treatment or therapeutic intervention. The event does not generally interfere with usual activities of daily living.

Moderate: A type of AE that is usually alleviated with specific therapeutic intervention. The event interferes with usual activities of daily living, causing discomfort but poses no significant or permanent risk of harm to the research patient.

Severe: A type of AE that interrupts usual activities of daily living, or significantly affects clinical status, or may require intensive therapeutic intervention.

7.3 Causality

The PI or designee must make the assessment of relationship to SP for each AE. The PI or designee should decide whether, in his or her medical judgment, there is a reasonable possibility that the event may have been caused by the SP. If there is no valid reason for suggesting a relationship, then the AE should be classified as "not related". Otherwise, if there is any valid reason, even if undetermined or untested, for suspecting a possible cause-and-effect relationship between the SP and the occurrence of the AE, then the AE should be considered "related". The causality assessment must be documented in the source document.

The following additional guidance may be helpful:

Related (definitely, probably, possibly related): The temporal relationship between the event and the administration of KB109 is compelling and/or follows a known or suspected response pattern to that product, and the event cannot be explained by the patient's medical condition, other therapies, or accident.

Not Related (definitely not, unlikely related): The event can be readily explained by other factors such as the patient's underlying medical condition, concomitant therapy, or accident and no plausible temporal or biologic relationship exists between the SP and the event.

7.3.1 Outcome Categorization

The outcome of AEs must be recorded during the course of the study in the eCRF. Outcomes are as follows:

- Fatal
- Not recovered/Not resolved
- Recovered/Resolved
- Recovered/Resolved with Sequelae
- Recovering/Resolving
- Unknown

7.3.2 Symptoms of the Disease Under Study

COVID-19 related symptoms of the disease under study should not be classified as TEAEs as long as they are within the normal day-to-day fluctuation or expected progression of the disease *not including hospitalizations* and are part of the clinical data of the disease that is being collected in the study; however, clinically significant worsening of the symptoms should be recorded as a TEAE (or SAE).

7.3.3 Clinical Laboratory and Other Assessments

A change in the value of a study assessment can represent an AE if the change is a clinically significant worsening from baseline. This includes abnormal assessments where there is a shift of a parameter from a normal value to an abnormal value, or a significant worsening of an already abnormal value. Clinical significance is defined as an abnormal study assessment that leads to a diagnosis or results in patient intervention such as further monitoring (excluding confirmatory repeat testing) or medical treatment.

Note: Medications allowed and used as part of SSC to acutely relieve symptoms of COVID-19 (see Section 6.6) will be captured in the Healthcare Utilization assessment.

If, at the end of the SP Intake Period, there are abnormal study assessments which were not present pre-treatment, the value observed closest to the start of study treatment should be used as

baseline. The PI or designee should decide, based on the above criteria and the clinical condition of a patient, whether a worsening of an abnormal study assessment is clinically significant and therefore represents an AE.

7.3.4 Pregnancy

All pregnancies are to be reported from the time informed consent is signed to the end of follow-up.

Any report of pregnancy for any female study patient must be reported within 24 hours to the Sponsor. Pregnant female patients must be withdrawn from the study.

Every effort should be made to gather information regarding the pregnancy outcome and condition of the infant. It is the responsibility of the PI to obtain this information within 30 calendar days after the initial notification and approximately 30 calendar days and 1-year post-partum.

Pregnancy complications such as spontaneous abortion/miscarriage or congenital abnormality are considered SAEs and must be reported as such. Note: an elective abortion is not considered an SAE.

In addition to the above, if the PI determines that the pregnancy meets SAE criteria, it must be reported as an SAE. The test date of the first positive urine human chorionic gonadotropin test or ultrasound result will determine the pregnancy onset date.

7.4 SAE Procedures

7.4.1 SAE definition

A SAE is defined as any untoward medical occurrence (whether considered to be related to KB109 or not) that at any dose fulfills at least one of the following criteria:

1. Results in death
2. Is life-threatening
   (*Note*: the term “life-threatening” in the definition of “serious” refers to an event in which the patient was at risk of death at the time of the event; it does not refer to an event which could hypothetically have caused death had it been more severe)
3. Requires inpatient hospitalization or prolongation of existing hospitalization
   (*Note*: hospitalizations which are the result of elective or previously scheduled surgery for pre-existing conditions, or which have not worsened after initiation of treatment,

should not be classified as SAEs. For example, an admission for a previously scheduled ventral hernia repair would not be classified as an SAE; however, complication[s] resulting from a hospitalization for an elective or previously scheduled surgery that meet[s] serious criteria must be reported as SAE[s]).

4. Results in persistent or significant disability/incapacity
5. Is a congenital anomaly/birth defect
   (*Note:* congenital anomaly/birth defect in offspring of patient taking KB109 regardless of time to diagnosis)
6. Is an important medical event
   (*Note*: important medical events that may not result in death, be life-threatening, or require hospitalization may be considered an SAE when, based upon appropriate medical judgment, they may jeopardize the patient and may require medical or surgical intervention to prevent one of the outcomes listed in this definition. Examples of such medical events include allergic bronchospasm requiring intensive treatment in an emergency room or at home; blood dyscrasias or convulsions that do not result in inpatient hospitalization; or the development of drug dependency or drug abuse)

7.4.2 SAE reporting procedures

All SAEs occurring from the time a patient signs informed consent until the end of the follow-up period must be reported to Medpace Clinical Safety within 24 hours of the knowledge of the occurrence.

To report SAEs, the site should complete the SAE form electronically in the electronic data capture (EDC) system for the study. When the form is completed, Medpace Safety personnel will be notified electronically by the EDC system and will retrieve the form.

If the event meets serious criteria and it is not possible to access the EDC system, send an email to Medpace Safety or call the Medpace Safety hotline (contact information listed below), and fax/email the completed back-up paper SAE form to Medpace within 24 hours of awareness. When the EDC system becomes available, the SAE information must be entered within 24 hours of the system becoming available.

Safety Contact Information: Medpace Clinical Safety
Medpace SAE reporting line – USA:
Fax: +1-866-336-5320 or +1-513-570-5196
e-mail: medpace-safetynotification@medpace.com
Telephone: +1-800-730-5779, dial 3 or +1-513-579-9911, dial 3

7.4.3 SAE Collection Timeframe

All SAEs (regardless of relationship to KB109) are collected from the time the patient signs the informed consent until the end of follow-up and must be reported to the Sponsor and CRO within 24 hours of the first awareness of the event (Section 7.4.2).

In addition, any SAE considered “related” to KB109 and discovered by the PI or designee at any interval after the study has completed must be reported to the CRO within 24 hours of the first awareness of the event (Section 7.4.2).

7.4.4 SAE Onset and Resolution Dates

The onset date of the SAE is defined as the date the event meets serious criteria. The resolution date is the date the event no longer meets serious criteria, the date the symptoms resolve, or the event is considered chronic. In the case of hospitalizations, the hospital admission and discharge dates are considered the onset and resolution dates, respectively.

In addition, any signs or symptoms experienced by the patient after signing the informed consent form, or leading up to the onset date of the SAE, or following the resolution date of the SAE, must be recorded as an AE, if appropriate.

7.4.5 Fatal Outcome

Any SAE that results in the patient’s death (i.e., the SAE was noted as the primary cause of death) must have ‘Fatal’ checked as an outcome with the date of death recorded as the resolution date. For all other events ongoing at the time of death that did not contribute to the patient’s death, the outcome should be considered ‘Not Resolved’, without a resolution date recorded.

For any SAE that results in the patient’s death or any ongoing events at the time of death, the action taken with the SP should be recorded as “dose not changed” or “not applicable” (if the patient never received SP).

7.4.6 Reporting to health authorities, independent ethics committee and investigators

The PI must comply with any applicable site-specific requirements related to the reporting of SAEs (and in particular deaths) involving his/her patients to the Independent Ethics Committee (IRB) that approved the trial. In accordance with ICH-GCP guidelines, the Sponsor will inform the PI of “findings that could adversely affect the safety of patients, impact the conduct of the

trial, or alter the IRB's approval/favorable opinion to continue the trial." In line with respective regulations, the Sponsor will inform the PI of AEs that are both serious and unexpected and are considered to be related to the administered product ("suspected unexpected serious adverse reactions"). The Sponsor will send appropriate safety notifications to relevant health authorities if required in accordance with applicable laws and regulations.

The PI should place copies of safety reports in the Investigator Site File (ISF). National regulations regarding safety report notifications to the PI will be taken into account. When specifically required by regulations and guidelines, the Sponsor will provide appropriate safety reports directly to the concerned IRB and will maintain records of these notifications. When direct reporting by the Sponsor is not clearly defined by national or site-specific regulations, the PI will be responsible for promptly notifying the concerned IRB of any safety reports provided by the Sponsor and of filing copies of all related correspondence in the ISF.

8 STATISTICAL CONSIDERATIONS

This section outlines the statistical analysis strategy and procedures for the study. The details of the statistical methods will be provided in a separate technical document or in the Statistical Analysis Plan (SAP).

8.1 Sample Size

This is an exploratory study; thus, no formal sample size or power calculations are employed. The sample size of 50 randomized patients (25 per group) is chosen for practical reasons.

8.2 Analysis Endpoints

The study endpoints for evaluation are listed in Section 3.2.

8.3 Analysis Set

8.3.1 Safety Analysis Set

The Safety Analysis Set will include all randomized patients. Patients will be included in the group based on the fact that whether or not they actually consumed any amount of KB109: patients who actually consumed any amount of KB109 will be in the SSC + KB109 group; otherwise patients will be in the SSC alone group. The study analysis will be based on the Safety Analysis Set unless otherwise specified.

8.4 Statistical Methods

This section describes the statistical methods for the clinical endpoints of the study. Methods related to microbiome endpoints and supportive analyses will be described in a separate document such as Statistical Analysis Plan (SAP) or technical report.

8.4.1 Patient Disposition

The frequency and percentage of patients in each population along with disposition (completed study, early termination, with breakdown for reasons for discontinuation) will be summarized overall and by group (SSC or SSC + KB109).

8.4.2 Demographics and Baseline Characteristics

Patient demographics (including age, gender, race, ethnicity, weight, height, and BMI) and baseline characteristics, medical history, and concomitant therapies will be summarized by group either by descriptive statistics or categorical tables.

8.4.3 Analyses of Endpoints of Interest

Adverse Events

Adverse events will be coded using the most recent version of Medical Dictionary for Regulatory Activities (MedDRA).

A TEAE is defined as any AE starts or worsens in severity on or after the first dose of SP is taken for SSC + KB109 group or on or after the randomization for SSC group. Adverse event summary tables will include all TEAEs, SP-related TEAEs, SAEs, TEAEs by severity, and TEAEs leading to product discontinuation. Summaries will be presented by MedDRA system organ class and preferred term using frequency counts and percentages by group and overall.

Other Endpoints

For the endpoints listed below, continuous data will be summarized by group and by visit using the following descriptive statistics: sample size, mean, standard deviation, median, minimum

value, and maximum value. Categorical and qualitative data will be summarized by group and by visit using frequencies and percentages.

- Measures of QOL
- Measures collected from the Healthcare Provider Wellness Visits
- Proportion of patients experiencing hospital admissions during the intake period and follow-up period (all cause, and COVID-19-related)
- Healthcare Utilization during the Intake Period and Follow-up Period
- Proportion of patients with oxygen saturation <95%
- Proportion of patients with oxygen saturation <98%
- Proportion of patients with temperature below 100.4 °F without an antipyretic medication

8.4.4 Extent of Exposure and Compliance

The cumulative exposure to SP, including duration of exposure and cumulative amount of SP received, will be summarized using descriptive statistics.

Intake information will be provided in a data listing.

9 SUPPORTING DOCUMENTS AND OPERATIONAL CONSIDERATIONS

9.1 Regulatory, Ethical, and Study Oversight Considerations

This study will be initiated only after all required documentation has been reviewed and approved by the respective IRB. The same applies for the implementation of changes introduced by amendments.

The PI will ensure that this study is conducted in full conformity with Regulations for the Protection of Human Patients of Research codified in 45 CFR Part 46, 21 CFR Parts 50 and 56, and/or the principles in the ICH E6 (R2) GCP guideline.

9.1.1 Informed Consent

Informed consent is a process that is initiated prior to the individual's agreeing to participate in the study and continues throughout the individual's study participation. Discussion of risks and possible benefits of participation will be provided to the patients. Consent forms will be IRB-approved, and the patient will be asked to read and review the document. The PI, or designee, will explain the research study to the patients and answer any questions that may arise. All patients will receive a verbal explanation in terms suited to their comprehension of the purposes, procedures, and potential risks of the study and of their rights as research patients. Patients will have the opportunity to carefully review the consent form and ask questions prior to completing. The rights and welfare of the patients will be protected by emphasizing to them that the quality of their medical care will not be adversely affected if they decline to participate in this study. The patients may withdraw consent at any time throughout the course of the study.

The ICF will be retained in the patient's records and a copy of the ICF will be provided to the patient.

If a protocol amendment is required, then the ICF may need to be revised to reflect the changes to the protocol. If the ICF is revised it must be reviewed and approved by the responsible IRB. A determination will be made regarding whether previously consented patients need to be re-consented.

9.1.2 Institutional Review Board/Ethics Committee Review

The protocol, ICF, and all patient materials will be submitted to the IRB for review and approval. A copy of the IRB approval letter must be supplied to the Sponsor prior to starting the study. Approval of both the protocol and the ICF must be obtained before any patient is enrolled. Any amendment to the protocol will require review and approval by the IRB before the changes are implemented to the study. All changes to the ICF will be IRB approved; a determination will be made regarding whether previously consented patients need to be re-consented.

A protocol change intended to eliminate an apparent immediate hazard may be implemented immediately provided that the Sponsor, the Contract Research Organization, and the IRB are immediately notified.

9.1.3 Safety Monitoring Plan

Safety oversight of the study will be under the direction of the study medical monitor, overseen by the Sponsor. Detailed information on the nature of safety data review will be described in a study-specific Safety Monitoring Plan (MP).

9.1.4 Quality Assurance and Quality Control

Quality Control (QC) procedures will be implemented beginning with the data entry system and data QC checks that will be run on the database will be generated. Any missing data or data anomalies will be communicated to the site(s) for clarification/resolution. Additional details may be found in the study-specific Data Management Plan.

Following written standard operating procedures, the monitors will verify that the clinical study is conducted and data are generated, documented (recorded), and reported in compliance with the protocol, the principles of GCP in ICH E6 (R2) and any applicable regulatory requirements.

The investigational sites will provide direct access to study-related sites, source data/documents, and reports for the purpose of monitoring and auditing by the Sponsor and inspection by local and regulatory authorities.

9.1.5 Direct Access to Source Data and Documents

Clinical site monitoring is conducted to ensure that the rights and well-being of human patients are protected, that the reported study date are accurate, complete, and verifiable, and that the conduct of the study complies with the currently approved protocol/amendment(s), with applicable regulatory requirements, and with GCP guidelines. The Sponsor or their designees will monitor the conduct of the study by monitoring visits and in-house data quality review. The PI will permit study-related monitoring, audits, IRB review and regulatory inspections. Direct access must be provided to the ICFs, eCRF and source documents/data, including progress notes, copies of laboratory and medical test results. The accuracy of the data will be verified by direct comparison with source documents.

9.1.6 Publication

The results of this clinical study may be published or presented at scientific meetings by the Sponsor and/or PI. If this is foreseen, the PI agrees to submit all manuscripts or abstracts to the Sponsor prior to submission. This allows the Sponsor to protect proprietary information and to

provide comments based on information from other studies that may not yet be available to the PI (as specified in the contract).

In accordance with standard editorial and ethical practice, the Sponsor will generally support the publication of multicenter trials only in their entirety and not as individual center data. In this case, a coordinating PI will be designated by mutual agreement.

Authorship will be determined by mutual agreement.

9.2 Administrative and Legal Obligations

9.2.1 Protocol Amendments

The protocol will be submitted to the IRB for review and approval prior to initiation of study. Any amendment to the protocol will require review and approval by the IRB before the changes are implemented to the study.

9.2.2 Halting of Study

SP administration may be halted if the PI or the Sponsor identifies any unexpected, significant, or unacceptable risk to patients upon ongoing review of aggregate safety data. The Sponsor will inform the PI(s) promptly should the study be halted. If SP administration is halted, the PI will promptly inform the IRB and provide reason(s) for halting.

9.2.3 Study Termination

This study may be suspended or prematurely terminated by the Sponsor if there is reasonable cause. Written notification documenting the reason for study suspension or termination will be provided by the Sponsor to the PI. If the study is prematurely terminated or suspended, the PI will promptly inform the IRB and will provide the reason(s) for the termination or suspension.

Circumstances that may warrant termination or suspension include, but are not limited to:

- Determination of unexpected, significant, or unacceptable risk to patients, as determined by the ongoing review of aggregate safety data by the Sponsor
- Site submits data that are consistently insufficient, incomplete, and/or unevaluable

If the study is suspended temporarily, the study may resume once concerns about safety, protocol compliance, and/or data quality are addressed and satisfy the Sponsor and/or IRB.

9.2.4 Study Documentation and Archive

The site and telemedicine provider will maintain appropriate medical and research records for this study, in compliance with the protocol, the principles of GCP in ICH E6 (R2), relevant standard operating procedures, and any applicable regulatory and institutional requirements, including for the protection of confidentiality of patients. The PI must make study data accessible to the Sponsor, to other authorized representatives of the Sponsor, and to the appropriate regulatory authority inspectors.

Source data are information, original records of clinical findings, observations, or other activities in a clinical study necessary for the reconstruction and evaluation of the study. Examples of these original documents and data records include, but are not limited to, hospital records, clinical and office charts, laboratory notes, memoranda, patients' questionnaires and diaries, pharmacy dispensing records, copies or transcriptions certified after verification as being accurate and complete, X-rays, and patient files and records kept at the pharmacy and at the laboratories involved in the clinical study.

9.2.5 Electronic Case Report Forms

Data collection is the responsibility of the centralized telemedicine provider and clinical study staff at the site under the supervision of the site PI. The PI is responsible for ensuring the accuracy, completeness, legibility, and timeliness of the data reported.

Clinical data will be entered directly into the eCRFs from the source documents. Data reported in the eCRF derived from source documents should be consistent with the source documents, or the discrepancies should be explained and captured in a progress note and maintained in the patient's official electronic study record.

Clinical data (including AEs, concomitant medications, and expected adverse reactions data) and clinical laboratory data will be entered into the EDC system. The data system includes password protection and internal quality checks, such as automatic range checks, to identify data that appear inconsistent, incomplete, or inaccurate.

9.2.6 Record Retention

No records will be destroyed without the written consent of the Sponsor, per the terms agreed upon in the Clinical Study Agreement (CSA). It is the responsibility of the Sponsor to inform the PI when these documents no longer need to be retained.

9.2.7 SP Control, Accountability, and Disposition

All used and unused SP materials, including the SP packaging (as applicable) will be sent back to the Sponsor or designee for reconciliation and destruction. SP reconciliation may take place virtually.

9.2.8 Confidentiality

The Sponsor affirms the patient's rights to protection against invasion of privacy and to follow ICH/GCP guidelines and other local regulations. The study protocol, documentation, data, and all other information generated will be held in strict confidence. Except for emergency or specialist care, no confidential information concerning the study or the data will be released to any unauthorized third party without prior written approval of the Sponsor.

The study monitor, other authorized representatives of the Sponsor, and/or representatives of the IRB may inspect all documents and records required to be maintained by the PI or designee, including but not limited to, medical records (office, clinic, or hospital) and pharmacy records for the patients in this study. The Sponsor requires the PI to permit access to such records in accordance with local laws.

The study patient's contact information will be securely stored for internal use during the study. At the end of the study, all records will continue to be kept in a secure location until records no longer need to be retained per the terms in the CSA and applicable regulatory requirements.

Study patient data provided to the Sponsor will not include the patient's contact or identifying information. Patients will be identified by an assigned unique patient identification number on eCRFs, SAE reports, and other documents submitted to the Sponsor or Sponsor designated representative.

9.2.9 Disclosure of Data

Details and terms on publication and data sharing will be specified in the CSA between the Sponsor and site.

9.3 Protocol Deviations

A protocol deviation is any non-compliance with the requirements of the clinical study protocol, GCP, or other study agreements. The non-compliance may be either on the part of the patient, the PI, or the study site staff. As a result of deviations, corrective actions are to be developed by

the site and implemented promptly. There will be no preapproved protocol deviations in this study.

These practices are consistent with the principles in ICH E6 (R2):

- 4.5 – Compliance with Protocol
- 5.1 – Quality Assurance and QC
- 5.20 – Non-compliance

Protocol deviations relating to individual patients are to be addressed in the patient's source documents and on the appropriate eCRF (as applicable) and reported to the Sponsor. Deviations that are not patient-specific (e.g., unauthorized use of an SP outside of the study) will be reported to the Sponsor in writing and a copy of the report will be filed in the study-specific trial master file. Protocol deviations must be sent to the IRB per their guidelines. The PI and study staff are responsible for knowing and adhering to their IRB requirements. Further details about the handling of protocol deviations will be included in study oversight documents and plans.

9.4 Research Use of Stored Human Samples and Data

- **Intended Use:** Samples and data collected under this protocol will be used to study the objectives described in Section 3.1.
- **Storage:** Access to stored samples will be limited. Samples and data will be stored using codes assigned throughout the study. Data will be kept in password-protected computers, access- and role-restricted database, and sample tracking systems. Only the site's delegated study personnel and Sponsor's study team members will have access to the stored samples and data.
- **Tracking:** Samples will be tracked using the Sponsor's (or Sponsor's designated sample storage vendor's) specified sample tracking system. Disposition at the completion of the study will be conducted as follows:
  - Clinical study data will be archived as described in the Data Management Plan
  - Samples collected through this study will be stored by the Sponsor beyond completion of the study up to a period of five years after approval of the clinical study report. Study patients who request destruction of samples will be notified of compliance with such request and all supporting details will be maintained for tracking. Once a sample has been analyzed, the sample is considered consumed and cannot be destroyed. Data associated with this consumed sample will remain in the analysis dataset.

9.5 Future Use of Stored Samples and Data Derived from Samples

With the patient's consent and as approved by IRBs, de-identified biological samples and data will be stored by the Sponsor or its designee. These samples and data could be used for the Sponsor's research and development activities. The Sponsor's researchers and its designees may also be provided with a code-link that will allow linking the biological samples with the phenotypic data from each patient, maintaining the de-identification of each patient's identity. The identification of each patient beyond linking of the existing samples and data will not be possible after the completion of the study.

When the study is completed, access to study data and/or samples (if available) will be provided to the Sponsor.

The Sponsor will store data derived from the analysis of these samples. After study completion, the de-identified, archived data will be stored by the Sponsor. The informed consent will include permission for the Sponsor to store or transfer data to repository vendors. De-identified or anonymized data may be published or shared with third parties.

10 REFERENCES

Antunes KH, Fachi JL, de Paula R, da Silva EF, Pral LP, Dos Santos AÁ, Dias GBM, Vargas JE, Puga R, Mayer FQ, et al. Microbiota-derived acetate protects against respiratory syncytial virus infection through a GPR43-type 1 interferon response. Nat Commun. 2019;10,3273.

Arpaia N, Campbell C, Fan X; Dikiy S, van der Veeken J, deRoos P, Liu H, Cross JR, Pfeffer K, Coffer PJ, Rudensky AY. Metabolites produced by commensal bacteria promote peripheral regulatory T-cell generation. Nature 2013;504(7480):451-5.

Bindels L, Delzenne N, Cani P, Walter J. Towards a More Comprehensive Concept for Prebiotics. Nature Reviews Gastroenterology & Hepatology 2015;12;303-310.

Bito H, Hamaguchi N, Hirai H. Safety evaluation of a newly-developed dietary fiber: resistant glucan mixture. J Toxicol Sci. 2016;41(1):33-44.

Burdock GA, Flamm WG. A review of the studies of the safety of polydextrose in food. Food Chem Toxicol.1999; 37(2-3):233-264.

Grabitske HA, Slavin JL. Gastrointestinal Effects of Low-Digestible Carbohydrates. Critical Reviews in Food Science and Nutrition 2009;49(4):327-360.

Gray M. Carbohydrate digestion and absorption: role of the small intestine. New Engl J Med. 1975;292.23:1225-1230.

Haak, BW, Littmann, ER, Chaubard, J-L, Pickard, AJ, Fontana, E, Adhi, F, Gyaltshen, Y, Ling, L, Morjaria, SM, Peled, JU, et al. Impact of gut colonization with butyrate producing microbiota on respiratory viral infection following allo-HCT. Blood 2018;131,2978–2986.

Ichinohe, T, Pang, IK, Kumamoto, Y, Peaper, DR, Ho, JH, Murray, TS, Iwasaki, A. Microbiota regulates immune defense against respiratory tract influenza A virus infection. Proc. Natl. Acad. Sci. U.S.A. 2011;108,5354–5359.

Johns Hopkins Coronavirus Resource Center 2020, https://coronavirus.jhu.edu/

Kau AL, Ahern PP, Griffin NW, Goodman AL; Gordon JI. Human nutrition, the gut microbiome and the immune system. Nature 2011;474(7351),327-336.

Rosshart SP, Herz J, Vassallo BG, Hunter A, Wall MK, Badger JH, McCulloch JA, Anastasakis DG, Sarshad AA, Leonardi I, Collins N, Blatter JA, Han S-J, Tamoutounour S, Potapova S, St. Claire MBF, Yuan W, Sen SK, Dreier MS, Hild B, Hafner M, Wang D, Iliev ID, Belkaid Y, Trinchieri G, Rehermann B. Laboratory mice born to wild mice have natural microbiota and model human immune responses. Science 2019;365(6452):EAAW4361.

Schirmer M, Smeekens SP, Vlamakis H, Jaeger M, Oosting M, Franzosa EA, ter Horst R, Jansen T, Jacobs L, Bonder MJ, et al. Linking the Human Gut Microbiome to Inflammatory Cytokine Production Capacity. Cell 2016;167,1125-1136.e8.

Smith PM, Howitt MR, Panikov N, Michaud M, Gallini CA, Bohlooly-Y M, Glickman JN, Garrett WS. The microbial metabolites, short-chain fatty acids, regulate colonic Treg cell homeostasis. Science 2013;341(6145),569-73.

Sommer F, Bäckhed F. The Gut Microbiota-Masters of Host Development and Physiology. Nat Rev. 2013; 11: 227-238.

Thaiss CA, Zmora N, Levy M; Elinav E. The microbiome and innate immunity. Nature 2016;535(7610),65-74.

Trompette A, Gollwitzer ES, Pattaroni C, Lopez-Mejia IC, Riva E1, Pernot J, Ubags N, Fajas L, Nicod LP, Marsland BJ. Dietary Fiber Confers Protection against Flu by Shaping Ly6c-Patrolling Monocyte Hematopoiesis and CD8+ T Cell Metabolism. Immunity. 2018;15,48(5),992-1005.e8.

Wils D, Scheuplein RJ, Deremaux L. Safety profile of a food dextrin: Acute oral, 90-day rat feeding and mutagenicity studies. Food and Chemical Toxicology. 2008; 46: 3254-3261.

Yokishawa Y, Kishimoto Y, Tagami H. Assessment of the safety of hydrogenated resistant maltodextrin: reverse mutation assay, acute and 90-day subchronic repeated oral toxicity in rats, and acute no-effect level for diarrhea in humans. J Toxicol Sci. 2013; 38(3): 459-470.
